# Single‐Target Pairing System (StarPair) for Large‐Scale Interrogation of Cell–Cell Interactions

**DOI:** 10.1002/advs.202513951

**Published:** 2025-12-12

**Authors:** Tianjiao Mao, Lang Nan, Miao Xu, Kehao Zeng, Yuchao Wang, Ziyu Han, Ho Cheung Shum

**Affiliations:** ^1^ Department of Mechanical Engineering The University of Hong Kong Pokfulam Road Hong Kong SAR 000000 China; ^2^ School of Instrument Science and Technology Xi'an Jiaotong University Xianning West Road Xi'an Shaanxi 710049 China; ^3^ Advanced Biomedical Instrumentation Centre Hong Kong Science Park Shatin, New Territories Hong Kong SAR 000000 China; ^4^ Department of Chemistry and Department of Biomedical Engineering City University of Hong Kong Tat Chee Avenue, Kowloon Hong Kong SAR 000000 China

**Keywords:** cell–cell interactions, droplets, functional immune cell screening, single‐target pairing

## Abstract

Coordination of cell populations through intercellular interactions is essential to the formation and function of living organisms. However, technologies enabling systematic dissection of cell–cell communications or manipulation of cell–cell interactions at the single‐cell resolution remain largely out of reach. To solve this, a single‐target pairing system, termed StarPair, is proposed, for the high‐throughput and high‐precision combination of desired single targets in droplets. How target buoyancy, target concentration, sorting parameters, droplet flow rates, and electric stimulation collectively determine the single‐target pairing performance is comprehensively explored. Upon optimization of these parameters, pairing efficiencies over 95% can be achieved with an operation frequency reaching one million single‐target pairs per 9.5 h for two‐target assembly. Leveraging StarPair, the immune cell‐cancer cell interactions are assessed between 4 × 10^5^ cell pairs and the NK‐92MI cells with high secretion capability are further enriched. It is envisioned that StarPair will open new perspectives for characterizing and manipulating cell–cell interactions at scale.

## Graphical abstract

This work presents a single‐target pairing system, StarPair, enabling cell–cell interaction studies by target combination in droplets. StarPair offers superior pairing efficiencies over 95% and operation frequencies of 10^5^ pairs per 9.5 h for two‐target pairing, allowing large‐scale interrogation of immune cell‐cancer cell interactions and precise functional immune cell screening, with potential applications in various intercellular interaction scenarios.

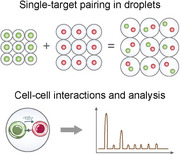

## 1 Introduction

Cells are indispensable for maintaining the structures and functions of complex multicellular organisms. The coordination of cellular behaviors through cell–cell interactions determines tissue homeostasis, organ development, and disease evolution.^[^ ^1^, ^2^ ^]^ For instance, cell circuits involving communications between different types of cells help stabilize the cellular phenotype and regulate organogenesis.^[^ ^3^, ^4^ ^]^ Interactions between the healthy cells and stromal cells profoundly impact tumor growth and progression.^[^ ^5^ ^]^ Therefore, interrogating cell–cell interactions is of vital importance for understanding various interaction processes and developing advanced therapeutics.

Short‐distance intercellular communications are generally divided into two categories: First, contact‐dependent interactions relying on ligand‐receptor interactions or gap junctions and other tunneling nanotubes to transfer molecules;^[^ ^6^ ^]^ second, secretion‐dependent interactions based on diffusion of signaling molecules between neighboring cells.^[^ ^7^ ^]^ To uncover the molecular underpinnings of these interactions, researchers have harnessed state‐of‐the‐art genetic engineering tools to modify cells and evaluate gene functions.^[^ ^8^, ^9^ ^]^ For instance, a protein suppressor in multiple sclerosis was identified via genetic forward screening of astrocyte‐microglia crosstalk.^[^ ^9^ ^]^ Genetic and cellular drivers for syncytium formation in severe acute respiratory syndrome coronavirus 2 (SARS‐CoV‐2) were discovered through large‐scale mutagenesis and genome‐wide knockout screening.^[^ ^10^ ^]^ cell–cell interactions have also been employed to develop therapeutic strategies.^[^ ^11^ ^]^ One typical example is the chimeric antigen receptor (CAR)‐T cell therapy. The synthetic receptors introduced to T cells permit their recognition and binding to tumor antigens, which subsequently induce T cell activation and cancer cell killing.^[^ ^12^, ^13^ ^]^ In these cases, library‐wide or even genome‐wide screening is demanded to systematically unveil how genetic perturbations in individual cells alter cell–cell communications or to discover individual cells of interest. To enable such screening, technologies for precisely controlling cell–cell interaction conditions (e.g., cell numbers, cell types, and external environment) are required. Importantly, the technologies should enable screening in a high‐throughput manner, as the input cell system often consists of millions of cells.^[^ ^13^ ^]^

In recent years, advanced imaging techniques, including high‐content imaging,^[^ ^14^, ^15^ ^]^ imaging flow cytometry,^[^ ^16^, ^17^ ^]^ and imaging mass cytometry,^[^ ^1^, ^18^ ^]^ have provided new opportunities for dissecting cell–cell interactions. These technologies can simultaneously assess multiple aspects of cells at the single‐cell resolution. However, they generally co‐culture cells in large vessels prior to analysis, thereby lacking defined intercellular interactions. Droplet microfluidics, which allows the generation of millions of picoliter droplet compartments, has enabled high‐efficiency processing and manipulation of single biological targets.^[^ ^19^, ^20^ ^]^ Facilitated by the droplet‐based co‐encapsulation method, single cells of two types can be paired in independent droplets to characterize their interactions.^[^ ^21^ ^]^ The droplets containing the desired cells can be further enriched by fluorescence‐activated droplet sorting techniques.^[^ ^22^, ^23^ ^]^ Droplet flow cytometry is also feasible for selecting targeting droplets by integrating double emulsion droplets or microgels with commercial flow cytometry.^[^ ^24^, ^25^ ^]^ Nevertheless, due to the limitation of the Poisson distribution, the efficiency for capturing two single cells in the same droplet is less than 5%, leading to a massive loss of cell samples.^[^ ^26^ ^]^ Microtrap/microwell‐based platforms have remarkably improved the pairing efficiency by hierarchically loading different batches of cells into the traps.^[^ ^27^, ^28^ ^]^ Cell manipulation can be implemented either passively through hydrodynamic force^[^ ^29^, ^30^, ^31^ ^]^ or actively through electric force,^[^ ^32^ ^]^ optic force,^[^ ^33^ ^]^ or operation of valves.^[^ ^34^ ^]^ However, these platforms cannot circumvent the exchange of molecules between individual traps or wells, thereby resulting in the cross‐contamination between neighboring cell pairs. By incorporating microtraps with droplets, the cells were automatically paired via droplet pairing and merging.^[^ ^35^, ^36^ ^]^ In this way, the secretion factors could be retained in the droplets without disrupting localized cell–cell communications. Nevertheless, the intrinsic disadvantages of microtrap/microwell‐based devices, which include limited throughput and difficult retrieval of interested cells, could not be overcome by incorporating the two techniques. Therefore, large‐scale single‐cell pairing is still an urgent and unfulfilled goal.

**Figure 1:**
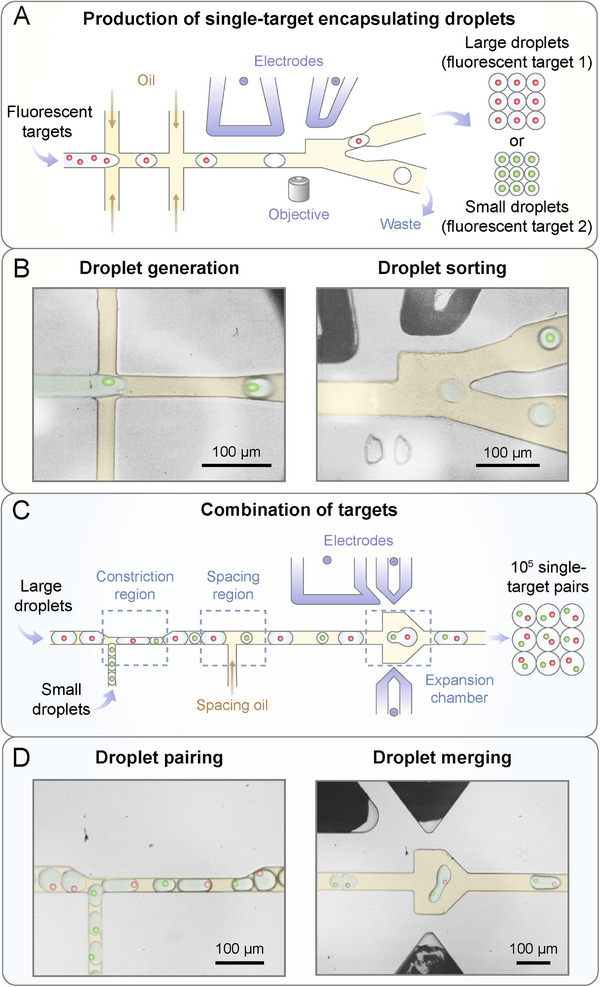
Workflow of single‐target pairing system (StarPair) for characterizing cell–cell interactions. A) Schematic and B) pictures showing the steps of generation and sorting of single‐target encapsulating droplets. C) Schematic and D) pictures showing the steps of pairing and merging of pre‐sorted droplets for producing single‐target pairs at scale.

To achieve this, we propose a single‐target pairing system named StarPair for large‐scale interrogation of cell–cell interactions. We integrate a combined droplet generation and sorting platform with a self‐synchronization‐based droplet pairing and merging platform and develop the two platforms into StarPair. All input fluorescent targets are first enriched in small and large droplets in a single‐target manner and then combined through merging of the single‐target encapsulating droplets (**Figure** **1**). We further profile interactions between NK‐92MI cells and K562 cells and enrich NK‐92MI cells with high‐level secretions, demonstrating the precision, scalability, and compatibility of StarPair in characterizing intercellular interactions.

## 2 Results

### 2.1 Dominating Factors in Single‐Target Pairing Performance of StarPair

**Figure 2:**
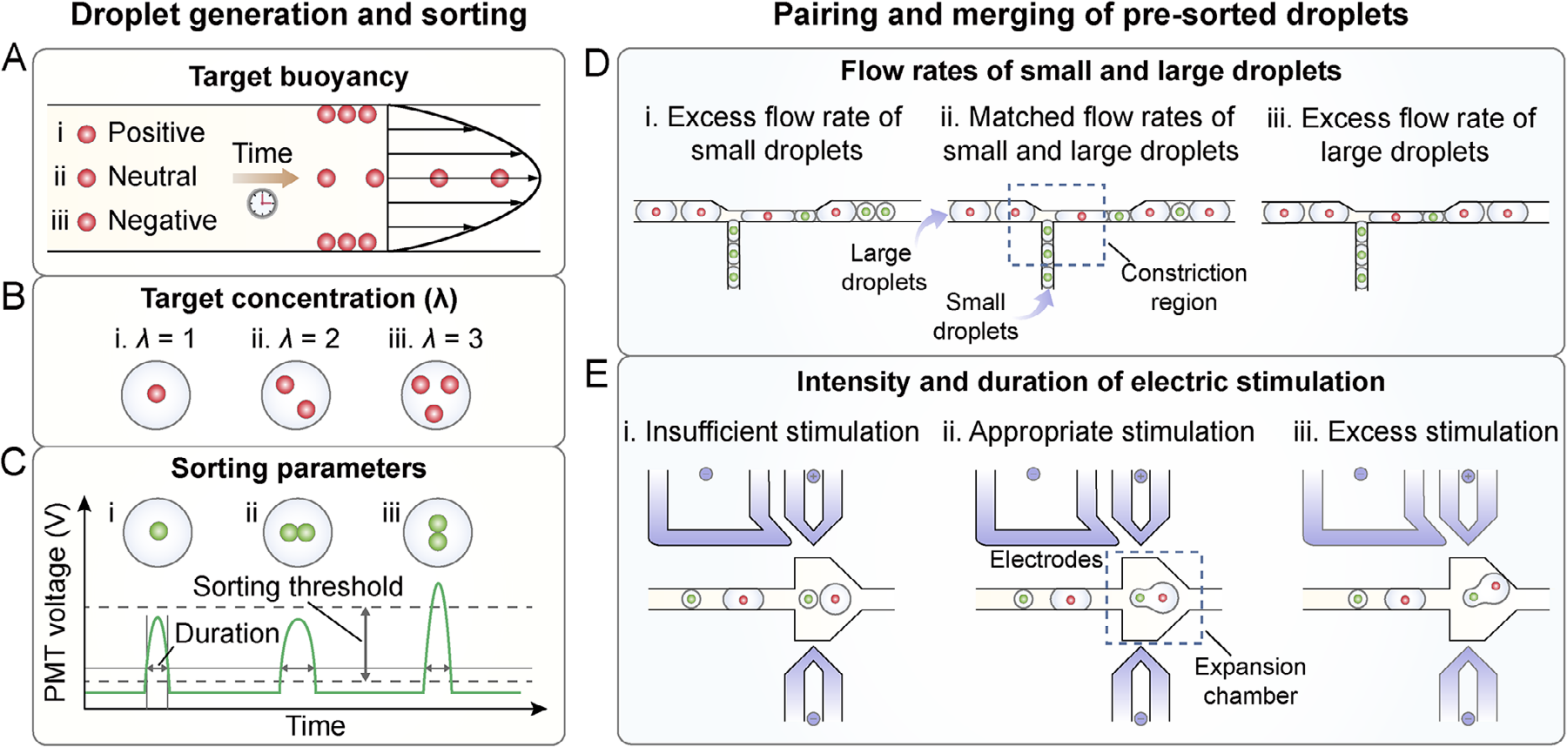
Schematic of parameters determining single‐target pairing performance of StarPair. A) Target buoyancy, B) target concentration, and C) sorting parameters impact the single‐target encapsulation efficiency in the process of droplet generation and sorting. D) Flow rate of two batches of droplets, as well as E) intensity and duration of electric stimulation, impact the combination efficiency of target‐encapsulating droplets in the process of pairing and merging of pre‐sorted droplets.

StarPair realizes single‐target pairing through two independent sets of steps: 1) droplet generation and sorting, and 2) pairing and merging of pre‐sorted droplets. The first set of steps determines the single‐target encapsulation efficiency in droplets, while the second set of steps determines the one‐to‐one combination efficiency of droplets carrying the desired targets. Therefore, the two sets of steps are equally important and collectively regulate the single‐target pairing performance. In the droplet generation and sorting process, three essential factors contribute to single‐target loading: target buoyancy, target concentration, and sorting parameters. Target buoyancy is determined by the density difference between the target and carrier fluid, with negative difference, zero difference, and positive difference leading to positive, neutral, and negative buoyancy, respectively (**Figure** **2**A). The inequality of the densities can cause target sedimentation or floatation in the medium during the droplet generation process, which further results in target aggregation and impedes single‐target encapsulation. According to Stokes’ law,^[^ ^37^ ^]^ the settling velocity *v* of targets can be described as

(1) $$v=\frac{2\left|\rho_{t}-\rho_{f}\right|R^{2}g}{9\mu }$$

where *ρ*_t_ and *ρ*_f_ represent the density of the target and the medium, respectively. *R*, *µ*, and g stand for the radius of the target, viscosity of the medium, and gravitational acceleration, respectively. From Equation 1, the settling velocity is positively correlated to the size of the target and the density difference. In the target encapsulation process, a density gradient medium, OptiPrep, is commonly added to adjust the density of the medium to prevent target sedimentation, as most of the biological targets present a higher density than the medium.^[^ ^38^ ^]^ Therefore, the concentration of OptiPrep is required to be optimized to attain neutral buoyancy of targets for stable encapsulation. Target concentration is another factor that affects single‐target loading (Figure 2B). The fraction of droplets carrying *x* targets *P* (*X* = *x*) obeys a Poisson distribution,^[^ ^26^ ^]^ which can be calculated as

(2) $$P\left(X=x\right)=\frac{\lambda^{x}\times e^{-\lambda }}{x!}$$

where *λ* refers to the average target number per droplet. From Equation 2, both the frequencies of droplets encapsulating single targets (*x* = 1) and of droplets encapsulating more than single targets (*x* > 1) increase as the input target concentration increases. Hence, appropriate target concentrations need to be selected to obtain more droplets with single targets while keeping the number of multiple‐target droplets within acceptable limits. Our droplet sorting system picks up the droplets encapsulating fluorescent targets based on the fluorescence intensity thresholds and the duration at a certain peak height (Figure 2C). For droplets encapsulating two targets or more, there is a higher chance of exhibiting a higher peak threshold or wider duration than single‐target droplets. Therefore, by adjusting the sorting parameters, a portion of the unwanted droplets can be ruled out, and the single‐cell encapsulation efficiency can thus be increased. In the process of pairing and merging pre‐sorted droplets, the two batches of droplets are first paired in the constriction region. The pairing efficiency is mainly affected by the flow rate of droplets (Figure 2D). Excess flow rate of small droplets or large droplets leads to a pairing ratio of small to large droplets higher than 1 or lower than 1, respectively. Only matched flow rates can result in 1‐to‐1 pairing of small and large droplets. After droplet pairing and spacing, droplet pairs are merged in the expansion chamber. The merging efficiency is concomitantly controlled by the intensity and duration of electric stimulation (Figure 2E). The electric field intensity depends on the amplitude at a given channel geometry, while the duration is regulated by the droplet flow rates. When insufficient stimulation is applied (low amplitude or high flow rate), droplet merging efficiency will decrease. Under excess stimulation, electrowetting of droplets to the channel wall and even the breakup of droplets can be induced. Therefore, the flow rates and electric field parameters should be optimized for enhancing the 1‐to‐1 droplet combination efficiency.

### 2.2 Generation and Sorting of Single‐Target Encapsulating Droplets

**Figure 3:**
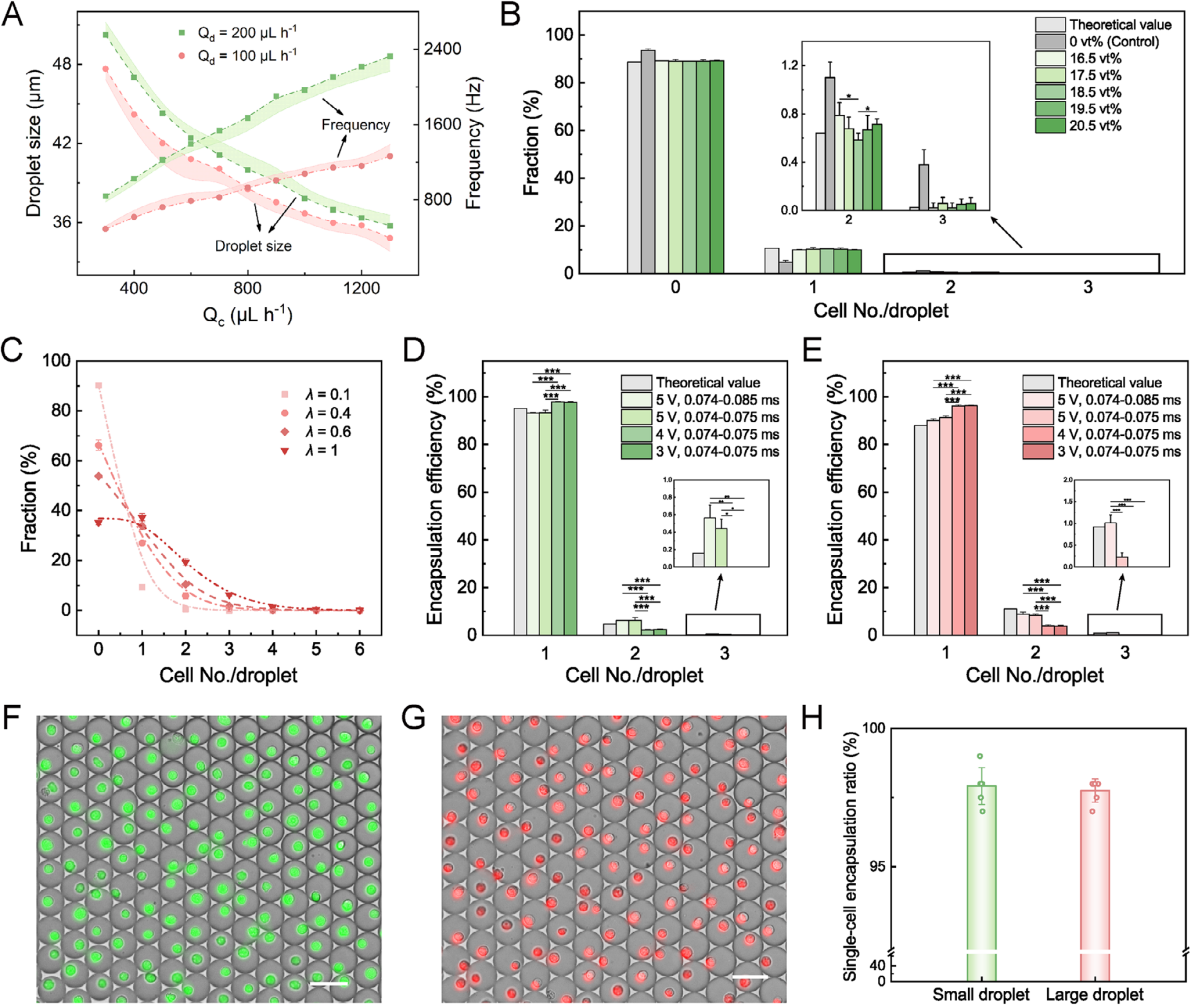
Droplet generation and sorting in StarPair. A) Droplet size and generation frequency at different flow rates of the first oil phase (Q_c_). The dashed lines and dash‐dotted lines represent droplet size and frequency, respectively. The flow rate of the dispersed phase (Q_d_) is set at 100 µL h^−1^ (pink) and 200 µL h^−1^ (green), and that of the second oil phase (spacing oil) is fixed at 2200 µL h^−1^. The shading areas depict the standard deviation. B) Fraction of droplets carrying different numbers of SP2/0 cells using a series of volume concentrations of OptiPrep (C_OptiPrep_) without sorting. For all these concentrations, *λ* = 0.12. The control group is not considered when calculating statistical significances, as OptiPrep is often added during cell encapsulation. C) Frequency of droplets carrying different numbers of cells using different SP2/0 cell concentrations (*λ*) without sorting. The dots represent the experimental results (n = 3, N > 1000) while dashed lines represent the theoretical values obtained from the Poisson distribution. C_OptiPrep_ = 18.5 vt%. D) Fraction of droplets encapsulating different quantities of SP2/0 cells after sorting with an appropriate cell concentration (*λ* = 0.1) but an inappropriate OptiPrep concentration (C_OptiPrep_ = 16.5 vt%) using different sorting parameters. E) Fraction of droplets encapsulating different quantities of SP2/0 cells after sorting with an appropriate OptiPrep concentration (C_OptiPrep_ = 18.5 vt%) but an inappropriate cell concentration (*λ* = 0.25) using different sorting parameters. Enrichment of single fluorescently stained SP2/0 cells in F) small and G) large droplets. H) Single‐cell encapsulation efficiency for small and large droplets (n = 5, N > 100). For different groups of sorting parameters, only the maximum peak threshold and the duration range are displayed, as the minimum peak threshold is fixed at 0.5 V. All the theoretical values in the figures are calculated according to Poisson equations.^[^ ^26^ ^]^ Statistical significances between the fractions in different groups with different OptiPrep concentrations or sorting parameters were analyzed by One‐way ANOVA (n = 3, N > 1000). ^*^ *p* < 0.05, ^**^ *p* < 0.01, and ^***^ *p* < 0.001. No significant difference is indicated if not annotated. Error bars depict SD. Scale bars: 50 µm.

As any pre‐mixing of different targets would cause the crosstalk between them, we designed a combined droplet generation and sorting platform, which is the first major component of StarPair, to produce the single‐target encapsulating droplets batch by batch (Figure 1A, B). Droplets are generated at the first cross junction, spaced by oil at the second cross junction, and then sorted at the branch channel area (Figure S1A, Supporting Information). The one‐step approach circumvents the potential instability of droplets induced by stepwise droplet generation and sorting processes. Since droplet pairing requires a size variance between two batches of droplets,^[^ ^39^ ^]^ we test the droplet size at different flow rates. The size is negatively correlated with the flow rate of the continuous phase (Q_c_) and positively correlated with that of the dispersed phase (Q_d_) (**Figure** **3**A). We select the Q_c_ of 1300 and 500 µL h^−1^ for creating small and large droplets (Q_d_ = 200 µL h^−1^), corresponding to the average droplet size of 35.7 and 44.5 µm, respectively. The uniform size distributions of the droplets are verified by the coefficient of variation lower than 1% (Figure S2, Supporting Information).

Using SP2/0 cells as the fluorescent targets, we quantitatively investigate how the three factors mentioned above (target buoyancy, target concentration, and sorting parameters) influence single‐target encapsulation in droplets. First, we resuspend SP2/0 cells with culture medium containing different concentrations of OptiPrep and measure the proportions of droplets carrying different numbers of cells (Figures 3B; S3, Supporting Information). When no OptiPrep is added, the single‐cell droplet fraction decreases from 10.6% (theoretical value) to 4.8%. With OptiPrep, the fraction of droplets containing single cells starts to rise. Within the concentration range of 16.5 to 20.5 vt%, the single‐cell droplet fraction presents a slight increase followed by a slight decrease, while the percentages of empty and double‐cell droplets show opposite trends. At 18.5 vt%, the individual fractions exhibit the minimum deviation from the theoretical values, indicating cells can achieve neutral buoyancy at this concentration. Using the optimized OptiPrep concentration, we evaluate the impact of cell concentration (*λ*) (Figures 3C; S4, Supporting Information). With the increase of *λ* from 0.1 to 1, the single‐cell droplet proportion drastically grows from 9.3% to 37.3%, but the multiple‐target droplet proportion (*P* (*x* > 1)) also surges from 0.45% to 27.5%. For all cell concentrations, the droplet fractions carrying specific cells display good agreement with the Poisson expectations, further demonstrating that the selected OptiPrep concentration (18.5 vt%) is appropriate. Next, we perform droplet sorting by employing four groups of sorting parameters with different peak thresholds and duration ranges to assess whether active sorting can reduce the quantity of multiple‐target droplets resulting from the inappropriate OptiPrep concentration (Figures 3D; S5, Supporting Information) and cell concentration (Figures 3E; S6, Supporting Information). At an OptiPrep concentration of 16.5 vt%, we find that the obtained proportion of single‐cell droplets is lower and the proportion of multiple‐cell droplets is higher than the corresponding theoretical values when using loose sorting parameters (5 V, 0.074‐0.085 ms). By narrowing the duration range down to 0.074–0.075 ms, the triple‐cell droplet fraction decreases a bit, but almost no change is observed for the double‐cell droplet fraction. When lowering the maximum peak threshold to 4 V, the fraction of double‐cell droplets significantly decreases while the fraction of single‐cell droplets significantly increases. However, further reduction of the peak threshold to 3 V shows no improvement in single‐cell droplet proportion. Similar results are discovered when sorting at a high cell concentration (*λ* = 0.25): narrowing the duration width and using a lower maximum sorting threshold decrease the fraction of droplets encapsulating triple and double targets, respectively, both of which are conducive to improving the single‐target encapsulation efficiency. These results demonstrate that although the input sample condition is not ideal, our sorting system can reduce the undesired types of droplets and elevate the ultimate single‐cell droplet fraction to a level even higher than theoretical predictions (Figure 3D, E). Nevertheless, a part of single‐cell droplets is inevitably eliminated when adopting stringent sorting parameters, leading to a decrease in the total amount of droplets collected (Figure S8, Supporting Information). Therefore, concomitant optimizations of OptiPrep concentration, cell concentration, and sorting parameters are essential for acquiring as many droplets as possible with a high single‐target encapsulation rate.

Upon coordinating the above three factors (detailed parameters see Table S1, Supporting Information), stable generation and sorting of droplets encapsulating single SP2/0 cells can be achieved for both small and large droplets (Movies S1 and S2, Supporting Information). At measured droplet generation frequencies of ≈2300 and 1200 Hz and appointed values of *λ*, we can obtain 5.4 × 10^5^ and 3.6 × 10^5^ small and large droplets per hour, respectively. The obtained droplets demonstrate single‐cell encapsulation efficiencies over 97% for both green and red fluorescently stained SP2/0 cells (Figure 3F–H). Besides, enrichment performances are consistent across four chips, validating the reproducibility of the platform (Figure S7A, B, Supporting Information). In addition to SP2/0 cells, we also explore whether our platform can reliably enrich other targets. At optimized conditions for each target category (Table S2, Supporting Information), our platform shows accurate single‐target enrichment with efficiencies above 96.5% for multiple types of cell targets including NK‐92MI cells, K562 cells, and NIH3T3 cells, an efficiency above 93% for a type of bacterial cell target, *Lactiplantibacillus plantarum*, and an efficiency above 97.5% for a type of unliving target, polystyrene beads (Figure S9, Supporting Information). The above results highlight the feasibility of our combined droplet generation and sorting platform to enrich diverse biological targets.

### 2.3 Pairing and Merging of Pre‐sorted Droplets Encapsulating Single Targets

Droplet merging is an effective strategy to assemble the desired components. Here, we modify the previously developed self‐synchronization‐based droplet pairing and merging platform^[^ ^39^ ^]^ and utilize it as the second major component of StarPair to realize the on‐demand pairing of different single targets by combining different batches of droplets encapsulating the targets (Figure 1C, D). Three major structures of the previous microfluidic design, constriction region, spacing region, and expansion chamber, are adopted for droplet pairing, spacing, and merging, respectively, while the channel geometries are adapted to accommodate the dimensions of our single‐target encapsulating droplets and filters are added in the droplet reinjection inlets to prevent clogging in the constriction region during long‐term merging process (Figure S1B, Supporting Information).

**Figure 4:**
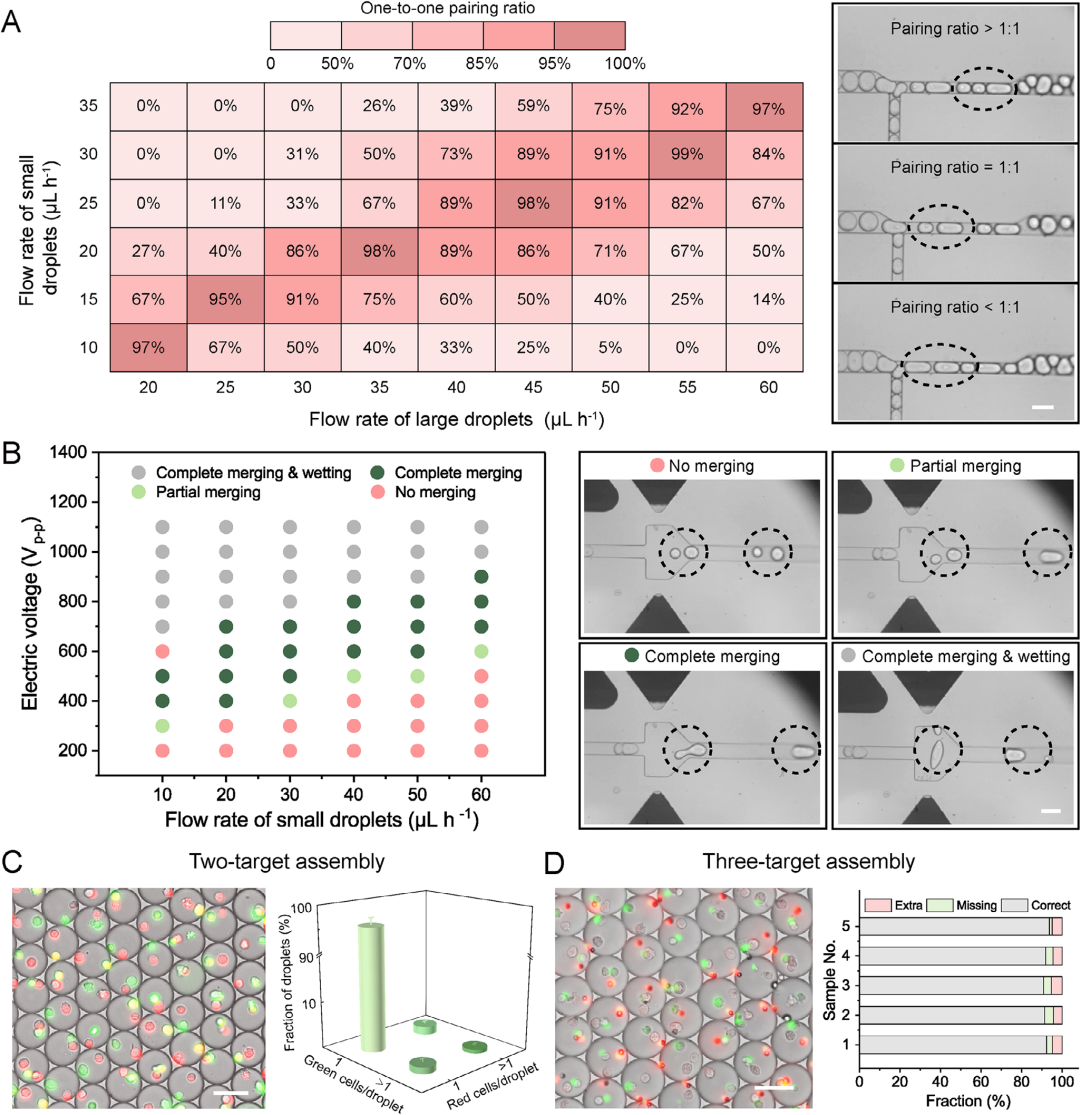
Pairing and merging of pre‐sorted droplets in StarPair. A) Left: One‐to‐one pairing ratios at different flow‐rate combinations of small and large droplets. Right: Microscope images showing droplet synchronization at different pairing ratios of small to large droplets. B) Left: Droplet merging changes with the electric voltage and flow rate of droplets. “No merging”, “Partial merging”, “Complete merging”, and “Complete merging & wetting” refer to a merging efficiency of 0%, 1–99%, 100%, and 100% (droplet breakup), respectively. The flow rates of large droplets are adjusted to achieve 1‐to‐1 pairing with small droplets at varying flow rates of small droplets. The frequency of the electric field is fixed at 30 kHz. Right: Microscope images showing different droplet merging scenarios. C) Two‐target assembly: Left: Microscope image of droplets loaded with a pair of SP2/0 cells (one red fluorescent and one green fluorescent target) through one round of combination of small and large droplets. Right: 3D bar chart showing the fraction of droplets encapsulating different numbers of green or red fluorescently stained cells. D) Three‐target assembly: Left: Microscope image of droplets loaded with single NK‐92MI cells (large green fluorescent target), K562 cells (large red fluorescent target), and polystyrene beads (small red fluorescent target) through two rounds of combinations of small and large droplets. Right: Stacked bar chart showing the fraction of droplets containing correct targets (two single cells and one single bead), missing targets, and extra targets. Scale bars: 50 µm.

As aforementioned, the droplet pairing and merging rates together determine the target combination efficiency. To explore the optimized flow rate conditions for precise pairing, we evaluate the one‐to‐one pairing ratios at different flow rate combinations of small and large droplets and depict the pairing efficiency map (**Figure** **4**A). For the tested flow rate window of large droplets from 20 to 60 µL h^−1^, a one‐to‐one droplet pairing efficiency above 95% can be achieved when the flow rate ratios of large to small droplets ranged between 1.67–1.83 (diagonal zone in Figure 4A). This indicates that we can flexibly adjust the flow rates to accurately pair the droplets as long as their flow rate ratio is kept within the correct range. When the flow rates approach the upper left or lower right corners of the pairing efficiency map, the pairing rates gradually decrease, and the pairing ratio of small to large droplets is increasingly greater than one or less than one, respectively. Subsequently, we investigate the impact of electric voltage and flow rate on the merging efficiency. In general, the droplet merging phase diagram can be divided into four regions, which are “No merging”, “Partial merging”, “Complete merging”, and “Complete merging & wetting” (Figure 4B). At fixed flow rates of droplets, the merging scenarios shift from “No merging” to “Complete merging & wetting” as the electric voltage increases, implying that a threshold electric field intensity is demanded to disrupt the droplet interfaces stabilized by surfactants and induce droplet coalescence. With the increment of the flow rate, the critical electric voltage triggering droplet fusion increases. This can be explained by the higher electric force needed for droplet merging within shortened electric stimulation durations caused by higher flow rates. It should be noted that although increasing the electric voltage can improve the merging efficiency, droplets might break up and exhibit electrowetting to the channel wall, probably jeopardizing the viability of cells encapsulated in the droplets. Therefore, the electric voltage value should be as low as possible while ensuring complete merging.

Upon optimizing the above crucial factors (detailed parameters see Table S1, Supporting Information), our self‐synchronization‐based platform demonstrates well‐organized droplet pairing and merging of pre‐sorted droplets: The two batches of droplets are automatically paired facilitated by the alternating droplet blocking at the T‐junction in the constriction region (Movie S3, Supporting Information); subsequently, droplets are temporarily separated in the spacing region and merged pairwise under an electric field in the expansion chamber (Movies S4 and S5, Supporting Information). At flow rates of 20 and 35 µL h^−1^ for small and large droplets, our platform can achieve a droplet combination efficiency of 98% and a frequency of ≈110 Hz. This suggests that 10^5^ droplet pairs can be precisely merged in 15 min. Owing to both the superior single‐target encapsulation efficiency as well as the efficiency of droplet pairing and merging, we attain an overall single‐target pairing efficiency of 95%, with only 5% of the merged droplets encapsulating extra cells (Figure 4C). Moreover, the pairing outcomes of our platform are highly consistent when using different devices (Figure S7C, Supporting Information). The above results demonstrate the ability of our system to assemble two targets. However, in some research scenarios, such as barcoding of cell–cell interactions and secretion analysis of cell–cell interactions, the assembly of multiple targets is necessitated. To demonstrate this, we first separately enrich the three targets in two batches of small droplets and one batch of large droplets; after that, we perform two rounds of merging consecutively to combine one batch of large and small droplets and then the merged droplets with another batch of small droplets to achieve three‐component assembly (Movies S6 and S7, Supporting Information). The assembled droplets are highly uniform in their sizes and manifest precise pairing of the targeting cells and beads (Figure 4D). For five samples, on average, 92.1% of the droplets show correct pairing, 3.2% are missing one type of cell or bead, and 4.7% encapsulate extra targets. To assess the compatibility of StarPair with the biological targets, we extract the cells from the merged droplets and test the cell viability. The cells from StarPair show no viability differences from those cultured in flasks (Figure S10, Supporting Information), revealing the great biocompatibility of StarPair.

### 2.4 Characterization of Turnaround Time of StarPair

**Figure 5:**
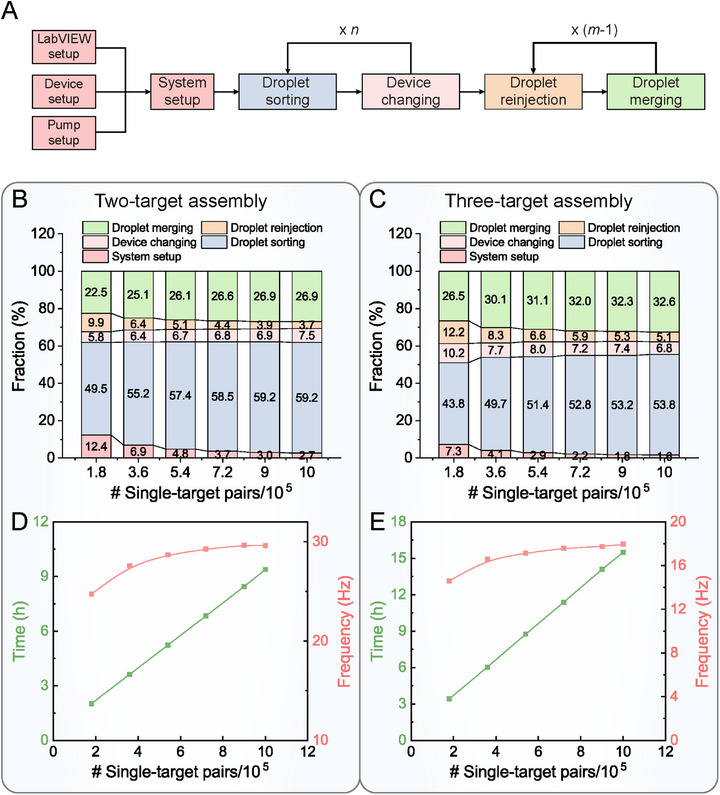
System manipulation and calculation of the turnaround time of StarPair. A) Experimental steps of StarPair. Droplet generation and sorting is abbreviated as droplet sorting, and droplet pairing and merging is abbreviated as droplet merging. *n* and *m* represent the number of rounds of droplet generation and sorting, and the number of target types to be combined, respectively. Cumulative percentage graphs showing the fraction of time consumption of each experimental step for B) two‐target assembly and C) three‐target assembly, respectively. System running and manual operation time are both included when calculating the turnover time, except for the time consumption for cell preparation. Line scatter graphs showing the turnaround time and corresponding frequency for obtaining different numbers of single‐target pairs for D) two‐target assembly and E) three‐target assembly, respectively.

In addition to the single‐target pairing accuracy, turnaround time or frequency is also a crucial indicator of the efficiency of a pairing system. For StarPair, the experimental procedures consist of system setup, droplet generation and sorting, device changing, droplet reinjection, and droplet pairing and merging (**Figure** **5**A). Among these steps, system setup, device changing, and droplet reinjection involve manual operations; droplet generation, sorting, pairing, and merging are automated with almost no human intervention needed once the system is settled. We calculate the time consumption of each step (detailed calculations see Methods) across a wide scale of target pairs. For both two‐target and three‐target assemblies, the percentage of manual operation time declines with increasing single‐target pair numbers (Figure 5B, C), leading to a slightly increased processing frequency. Altogether, StarPair is able to deliver 10^6^ pairs per 9.5 and 15.4 h for the two‐target and three‐target combination, corresponding to a frequency of ≈30 and ≈18 Hz, respectively (Figure 5D, E). Since droplet manipulation steps (generation, sorting, pairing, and merging) account for more than 80% of time consumption in most cases, the assembly frequency can be further improved by regulating the flow rates. Moreover, by adding a third channel in the constriction region for reinjecting an additional batch of droplets, the combination of three targets is possible with just one chip, and the time for droplet merging can be cut in half.^[^ ^39^ ^]^

### 2.5 Immune Cell‐Cancer Cell Interactions and Screening of Immune Cell Secretion

**Figure 6:**
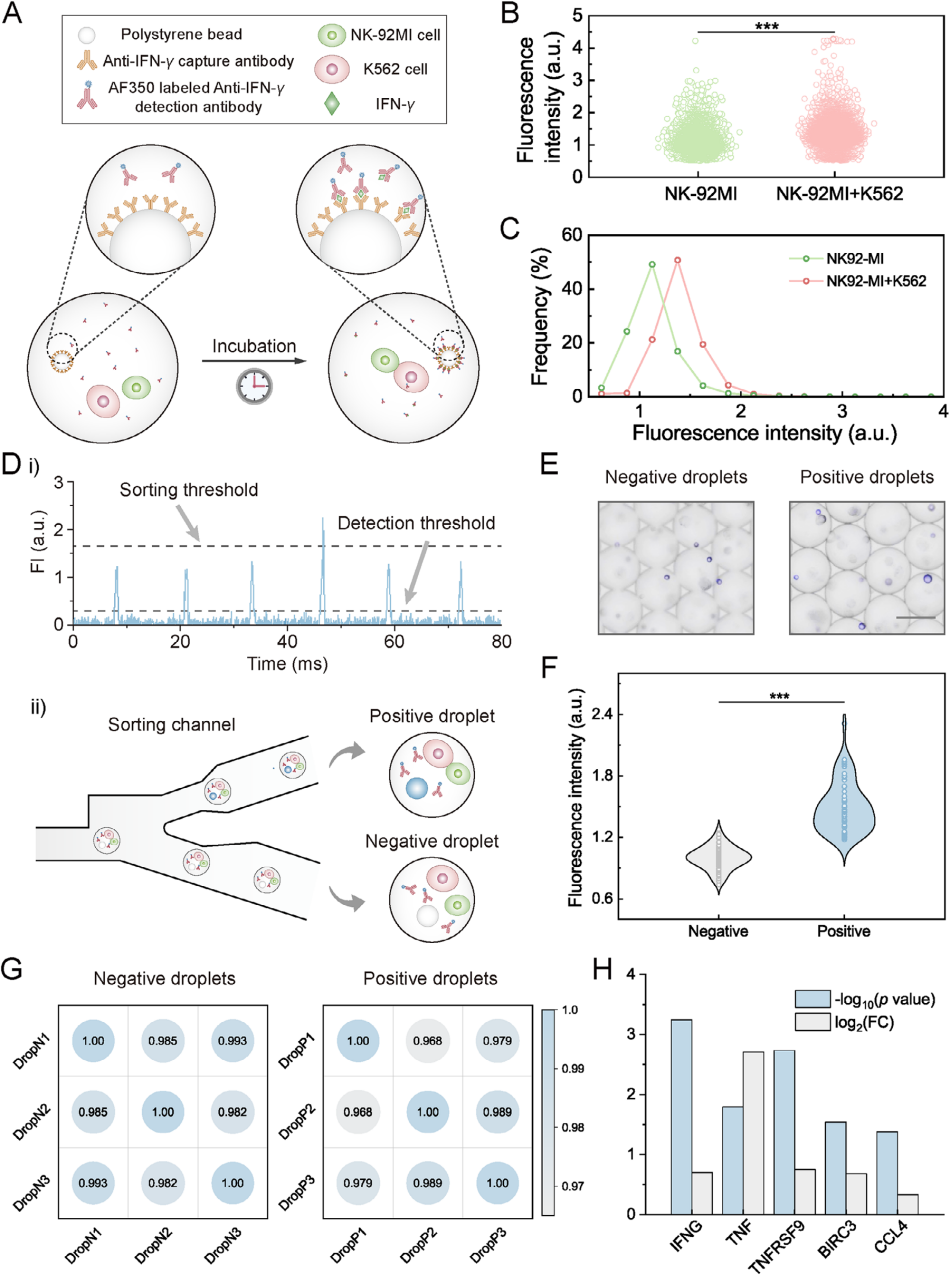
Characterization of NK‐92MI and K562 cell interactions and enrichment of NK‐92MI cells with high secretion capability. A) Schematic of the detection principle and key elements included in the assay. B) Fluorescence‐based screening of droplets. At least 15000 droplets are analyzed for both the NK‐92MI group and the NK‐92MI + K562 group. C) Line scatter graph showing the distribution of fluorescence intensity of droplets in two groups. D) Fluorescence‐based collection of positive and negative droplets. i) Real‐time fluorescence intensity (FI) of flowing droplets. ii) Droplets are sorted into the upper microfluidic channel if their PMT voltages exceed the sorting threshold (positive droplets). Otherwise, they are collected into the lower channel (negative droplets). E) Microscope images of negative and positive droplets. Scale bar: 50 µm. F) Violin plot depicting the fluorescence intensity of droplets. Kernel smooth is used for plotting. At least 60 droplets are analyzed for each group. G) Pearson correlation in gene expression of cells encapsulated in negative and positive droplets. “DropN1”, “DropN2”, and “DropN3” stand for three replicates of negative droplets. “DropP1”, “DropP2”, and “DropP3” stand for three replicates of positive droplets. H) Up‐regulated gene expressions obtained from RNA sequencing analysis that are related to NK‐92MI cell activation for cells in positive droplets compared with those in negative droplets. TNF, TNFRSF9, and CCL4 represent tumor necrosis factor, receptor superfamily member 9, and chemokine (C‐C motif) ligand 4, respectively. Statistical comparisons of the fluorescence intensity between NK‐92MI and NK‐92MI + K562 groups or negative and positive droplet groups were performed using Student's *t*‐test. ^***^ *p* < 0.001.

Chimeric antigen receptor‐based therapy is one of the milestones of cancer immunotherapy,^[^ ^31^, ^40^ ^]^ which isolates immune cells (e.g., T cells and natural killer (NK) cells) from the patient, genetically modifies the cells, and then infuses them back into the patient.^[^ ^41^ ^]^ Functional screening of the cell library is an indispensable step to reflect the antigen‐specificity and cytotoxicity of the cells.^[^ ^12^ ^]^ However, conventional pooled methods mask the cellular heterogeneity, rendering them unsuitable for discovering individual cells of interest. StarPair, which allows the generation of millions of droplets encapsulating single‐cell pairs, facilitates the identification of desirable cells at the single‐cell level. By utilizing StarPair, we create 4 × 10^5^ single‐cell paired droplets for surveying the interactions between single NK‐92MI cells and K562 cells. We design a droplet‐based bead immunoassay to measure the IFN‐*γ* production level of NK cells upon interacting with the cancer cells, as IFN‐*γ* is among the most common cytokines secreted by activated NK cells to combat tumor cells or invasive viruses.^[^ ^42^, ^43^ ^]^ The working principle of the immunoassay is: The produced IFN‐*γ* proteins are confined in independent droplets and are then captured on the surface of capture antibody‐coated beads for fluorescence‐based detection (**Figure** **6**A).

Before interrogating the IFN‐*γ* secretion features of single cells, we use standard IFN‐*γ* proteins to assess the assay feasibility. The bead immunoassay is first validated in bulk with an observed limit of detection of 1.28 ng mL^−1^ (Figure S11, Supporting Information). The average production of IFN‐*γ* per cell triggered by NK cell‐cancer cell interactions is ≈330 ng mL^−1^ (Figure S12A, B, Supporting Information). Based on this concentration level, we examine the fluorescence profiles of both IFN‐*γ* (+) and IFN‐*γ* (‐) groups by microfluidic fluorescence‐based screening (Figure S12C, Supporting Information). The IFN‐*γ* (+) group illustrates significantly improved fluorescence intensity compared with the IFN‐*γ* (‐) group (Figure S12D, Supporting Information). By setting a minimum sorting threshold at 1.49, we are able to accurately sort all single‐bead encapsulating droplets in the IFN‐*γ* (+) group while excluding 99.5% of IFN‐*γ* (‐) droplets into the lower waste channel (Figure S12E, F and Movies S8, S9, Supporting Information). Upon verifying the reliability of droplet‐based bead immunoassay and the fluorescence‐based screening system, we evaluate the IFN‐*γ* production induced by NK‐92MI‐K562 cell interactions at the single‐cell resolution systematically. We perform high‐throughput fluorescence scanning of all droplets after droplet assembly for 12 h to leave sufficient time for cell–cell interactions and the capture and detection of antigens, since the overall FI and the proportion of fluorescent beads remain almost unchanged after 8 h (Figure S13, Supporting Information). A control group is also established by pairing the NK‐92MI cells with the capture antibody‐modified beads but without the K562 cells (Figure S14, Supporting Information). A significant difference in fluorescence intensity between the two groups is demonstrated, with droplets in the NK92‐MI + K562 group presenting a higher average FI than the NK92‐MI group (1.39 vs 1.12) (Figure 6B, C), revealing the increased IFN‐*γ* production of NK cells through interacting with K562 cells.

Immune cells with enhanced cytokine secretion ability and cytotoxicity have been reported to present strengthened anti‐tumor efficacy.^[^ ^43^ ^]^ To showcase the capability of our strategy to enrich the NK‐92MI cells possessing high‐level IFN‐*γ* secretion, we dielectrically separate the droplets in the NK92‐MI + K562 group into two batches according to the fluorescence signals of individual droplets. By setting the minimum sorting threshold at 1.65, we collect the positive droplets ranked in the top 10% from the upper channel and the remaining negative droplets from the lower channel (Figure 6D; Movie S10, Supporting Information). Fluorescent images and fluorescence intensity analysis illustrate brighter beads in the positive droplets in comparison with the negative ones, confirming the accuracy of our approach (Figure 6E, F). To further explain the secretion differences of NK‐92MI cells in the two droplet groups at the molecular level, we disrupt the emulsion and extract the total RNA of cells for RNA sequencing. We first demonstrate that there is no transcriptional drift over the droplet manipulation process (Figure S15, Supporting Information). We then analyze the transcriptional profiles of cells in both sorted and waste samples. Pearson correlation coefficient for the three replicates reaches above 0.95, indicating that the assay is highly reproducible (Figure 6G). IFN‐*γ* gene expression in the positive cell population is significantly elevated relative to the negative cell population (‐log_10_(*p* value) = 3.24, log_2_(FC) = 0.70) (Figure 6H). Except for IFN‐*γ*, we also find other genes up‐regulated in the positive group, such as TNF, TNFRSF9, and CCL4 (Figure 6H). These genes have been recognized as essential markers associated with the effector immune response of NK cells.^[^ ^44^, ^45^ ^]^ The RNA sequencing results are further validated by qPCR (Figure S16, Supporting Information). Interestingly, although a 2.8‐fold and 602.6‐fold enrichment for granzyme B (GZMB) and colony‐stimulating factor 2 (CSF2), respectively, is identified when comparing the bulk co‐culture of NK‐92MI cells and K562 cells with bulk NK‐92MI cells (Figure S17, Supporting Information), the NK‐92MI cells in two droplet groups show no expression differences in cytolysis‐related genes.^[^ ^46^ ^]^ A possible explanation for this is that IFN‐*γ* release and the cytolytic status of NK cells may be independently regulated or temporally different.^[^ ^47^, ^48^ ^]^ Further screening of secretion proteins (e.g., GZMB) can be performed to interrogate the cytotoxic activity of NK cells when interacting with tumor cells and determine the correlation between the two primary functions of NK cells.

Overall, we propose a valuable method for characterizing NK‐92MI cell‐K562 cell interactions and enriching the NK‐92MI cells with enhanced cytokine secretion features at a large scale by adopting StarPair and the droplet‐based IFN‐*γ* immunoassay.

## 3 Discussion

Cell–cell interactions, which are involved in a wealth of biological processes, play crucial roles in the maintenance of the structure and function of multicellular organisms.^[^ ^1^, ^2^ ^]^ Systematic dissection and regulation of cell–cell communications at the single‐cell resolution anticipates a system capable of offering well‐defined cell–cell interaction conditions with distinguished throughput. Nevertheless, the majority of existing technologies lack rigorous control over the interaction parameters,^[^ ^16^, ^18^ ^]^ with the few available ones facing the dilemma of high pairing efficiency and high throughput.^[^ ^25^, ^34^, ^35^ ^]^ To fill this technical gap, we develop a single‐target pairing system called StarPair to enable precise cell pairing in independent microdroplets at a large scale. Key factors that influence the single‐target pairing efficiency, including target buoyancy, target concentration, sorting parameters, flow rates, and intensity and duration of electric stimulation, are extensively investigated. By optimizing these factors, we demonstrate an overall pairing efficiency of 95% and 92% and a manipulation frequency of > 25 and > 15 Hz for two‐component assembly and three‐component assembly, respectively. Although microtrap/microwell‐based platforms are able to combine two targets with good pairing efficiency, usually up to 70%, their throughputs are limited to only several thousand.^[^ ^27^, ^49^ ^]^ Nanovial‐based approaches can pair cells and measure secretions in a high‐throughput manner, but present relatively low pairing efficiency and lack control over the number of targets paired.^[^ ^50^, ^51^ ^]^ In comparison with these strategies, our StarPair system not only illustrates a superior two‐target pairing efficiency of 95% but can also supply million‐scale cell pairs within several hours. With StarPair, we also succeed in pairing three targets (Figure 4D), which is challenging to accomplish using traditional microtrap‐ or nanovial‐based methods. A combination of four targets or above is also possible by repeating the merging process or adding more channels for droplet reinjection. However, increasing rounds of merging will increase the reinjection errors due to droplet coalescence,^[^ ^52^ ^]^ while employing multiple reinjection channels places a higher demand on the regulation of flow rates and electric stimulation. Compared with other droplet‐based microfluidic systems,^[^ ^53^ ^]^ StarPair shows better pairing performance (92% vs 63% for three‐target assembly). StarPair also exhibits better performance in the assembly speed, displaying a 2‐fold and 5‐fold increase for assembling two and three targets, respectively. Comparisons between StarPair and currently available systems for single‐target pairing have been summarized in Table S3 (Supporting Information). One limitation of StarPair is that it requires relatively complex experimental setups, such as optical and electric modules, hence limiting it to laboratory use at the current stage. Concomitantly, a comparatively high degree of expertise is demanded for system operation.

Owing to the diversity and feasibility of droplet manipulations, StarPair can be easily integrated with other droplet‐based systems (e.g., fluorescence‐activated droplet sorting system, pico‐injection system, and droplet merging system) and various types of assays (e.g., immunoassay and polymerase chain reaction) for scores of applications. In this work, we showcase the advantages of StarPair by virtue of a biomedical application with common interests. We combine StarPair with a droplet‐based bead immunoassay and a fluorescence‐activated droplet sorting platform to fulfill the interrogation of the interactions between NK‐92MI cells and K562 cells of 4 × 10^5^ cell pairs. The IFN‐*γ* secretion of NK‐92MI cells upon interactions with K562 cells is quantitatively characterized, and the NK‐92MI cells with high secretion levels are enriched, with the system accuracy validated by the fluorescence intensity profiles, RNA‐sequencing, and qPCR. Such a high‐throughput approach for identifying individual NK cells of interest is reported for the first time and is demanded for functional cell screening in developing immune cell therapies. Currently, this approach can only select NK‐92MI cells according to IFN‐*γ* secretion. By changing the capture and detection antibodies, the secretion level of different target proteins (e.g., GZMB) can be measured to comprehensively characterize the function of NK cells. Aside from interrogating cell–cell interaction and secretion, our system also shows great prospects in other types of cell–cell interaction studies (Figure S18, Supporting Information). For instance, cell–cell fusion could be achieved by introducing poly(ethylene glycol) into paired cells in droplets.^[^ ^27^ ^]^

## 4 Conclusion

In summary, we describe a single‐target pairing system (StarPair) able to deliver single‐cell paired droplets at a million scale. The great potential of StarPair to investigate cell–cell interactions is demonstrated by the high‐throughput profiling of immune cell‐cancer cell interactions. Therefore, StarPair is well‐positioned as a promising tool for elucidating intercellular communication and expanding our knowledge of various physiological and pathological processes.

## 5 Experimental Section

### Materials and Reagents

HFE‐7500 (F051243) was purchased from Fluorochem. Neat fluorosurfactant (008‐FluoroSurfactant‐1G) was purchased from RAN Biotechnologies. OptiPrep density gradient medium (D1556‐250ML) and 1*H*,1*H*,2*H*,2*H*‐Perfluoro‐1‐octanol (370533) were purchased from Sigma‐Aldrich. Streptavidin‐coated fluorescent beads (purple, SVFP‐6062‐5) were purchased from Spherotech. CellTracker Red CMTPX dye (C34552), Calcein AM (C3100MP), cell viability imaging kit (R37610), anti‐IFN‐*γ* monoclonal antibody (2G1, M700A), biotinylated anti‐IFN‐*γ* monoclonal antibody (B133.5, M701B), Alexa Fluor 350 antibody labeling kit (A20180), and human IFN‐*γ* recombinant protein (300‐02‐20UG) were purchased from Thermo Fisher Scientific.

### Investigation of Parameters Affecting Single‐Target Pairing Performance

To investigate the influence of OptiPrep concentration on single cell encapsulation efficiency, SP2/0 cells (ATCC, CRL‐1581) were resuspended with 80 µL of culture medium supplemented with different concentrations of OptiPrep medium (0, 16.5, 17.5, 18.5, 19.5, and 20.5 vt%). Different concentrations (*λ* = 0.1, 0.4, 0.6, and 1) of cells were used when characterizing the effect of *λ*. To study the impact of sorting parameters, an inappropriate OptiPrep concentration (C_OptiPrep_ = 16.5 vt%) and an inappropriate cell concentration (*λ* = 0.25) were separately used. At the inappropriate OptiPrep concentration condition, a low cell concentration (*λ* = 0.1) was employed. Normal sorting parameters (0.5–5 V, 0.074–0.075 ms) were used for the first 70 µL of cells, and adapted sorting parameters were used for the last 10 µL of cells. At the inappropriate cell concentration condition, an optimized OptiPrep concentration (C_OptiPrep_ = 18.5 vt%) was employed. Adapted sorting parameters were employed for all 80 µL of cells. For the measurement of the 1‐to‐1 pairing ratio at different flow rate combinations of small droplets (10–35 µL h^−1^) and large droplets (20‐60 µL h^−1^) and the merging efficiency at different flow rates of small droplets (10–60 µL h^−1^) and electric voltages (200–1400 V_p‐p_), videos of droplets were recorded 30 s post changing the parameters and at least 100 droplet pairs were analyzed for each condition.

### Optimized Generation, Sorting, Pairing, and Merging of Single‐Target Encapsulating Droplets Using StarPair

To prepare cells for fluorescence‐activated sorting, cells were stained with CellTracker Red CMTPX dye (working concentration: 2 µm) or Calcein AM (2 µm) to gain red or green fluorescence, respectively. All fluorescent targets were resuspended in OptiPrep‐supplemented complete culture medium. The cell or bead solution and HFE‐7500 supplemented with 2 wt.% fluorosurfactant were used as the dispersed phase and continuous phase, respectively. By regulating the flow rates of the oil, droplets of different sizes were generated. For the small droplets, the flow rates of cell suspension, oil, and spacing oil were set at 200, 1300, and 2200 µL h^−1^, respectively; for the large droplets, the corresponding flow rates were set at 200, 500, and 2200 µL h^−1^, respectively. Different cell concentrations and sorting parameters were employed for small droplets (*λ* = 0.12; 0.5–4.5 V, 0.074–0.075 ms for the last 10 µL of cells) and large droplets (*λ* = 0.15; 0.5–4 V, 0.074–0.075 ms for the last 10 µL of cells) to achieve optimized sorting performance. Subsequently, the large and small droplets were reinjected into the merging device, where batches of droplets were synchronized, spaced, and merged successively.^[^ ^39^ ^]^ For the two‐component combination, the flow rates for the small droplets, large droplets, and spacing oil were set at 20, 35, and 200 µL h^−1^, respectively. The electric voltage to induce merging was 600 V_p‐p_. For the three‐component combination, the flow rates in the first round of merging were the same as in the two‐component combination, and the flow rates in the second round of merging for the small droplets, merged droplets, and spacing oil were set at 20, 55, and 300 µL h^−1^. The electric voltages in two rounds of merging were 600 V_p‐p_ and 650 V_p‐p_. To investigate the viability of cells after rounds of droplet manipulations, cells were stained with Calcein AM (2 µm) to generate and sort both small and large droplets encapsulating single cells to prevent confusion with the color of dyes in the cell viability imaging kit. After merging, droplets were treated with the demulsifier (1*H*,1*H*,2*H*,2*H*‐Perfluoro‐1‐octanol). The released cells were processed with the kit, with blue and red fluorescence representing living and dead cells, respectively.

### System Operation of StarPair and Calculation of Turnaround Time

System operation includes five aspects: system setup, droplet sorting, device changing, droplet reinjection, and droplet merging. System setup refers to the mounting of the combined generation and sorting device, configuration of the pumps and syringes, and startup of LabVIEW software and the amplifier, for which 15 min were enough. The generation and sorting devices should be hydrophobic throughout the droplet production process to prevent droplet coalescence. To this end, devices were changed after producing ≈160 µL of droplets. At the flow rates used for droplet generation and sorting, ≈5.4 × 10^5^ single‐target encapsulating small droplets and 3.6 × 10^5^ large droplets could be obtained per round (per hour and per device). Since the volume of small droplets was about half that of the large droplets, to facilitate droplet reinjection without reinjecting a lot of oil, the same rounds of sorting of small and large droplets were performed, although the number of sorted small droplets was higher than large droplets. The self‐synchronization‐based microfluidic platform was used for droplet merging after the completion of sorting. The time required for changing either the sorting device or the merging device each time was 7 min. Droplet reinjection was conducted at a low refill flow rate of 500 µL h^−1^ to prevent droplet coalescence. The tubing was placed vertically for ≈10 min to allow the oil to sink to the forefront end of the tubing. Then the pump was started to remove the oil before inserting the tubing into the merging device. This process took ≈10 min. Droplet synchronization and merging were then conducted at a frequency of 110 Hz.

The turnaround time can be calculated by adding the time consumed by the above steps, which can be described as

(3) $$t_{\mathrm{total}}=t_{\mathrm{setup}}+t_{\mathrm{sort}}+t_{\mathrm{change}}+t_{\mathrm{refill}}+t_{\mathrm{merge}}$$

where *t*_setup_, *t*_sort_, *t*_change_, *t*_refill_, and *t*_merge_ refer to the time cost for system setup, droplet generation and sorting, device changing, droplet reinjection and oil removing, and droplet pairing and merging, respectively. Among them, *t*_setup_ is a fixed value, which is *t*_setup_ = 15 / 60 h = 0.25 h. *t*_sort_ can be calculated by

(4) $$t_{\mathrm{sort}}=\frac{N_{P}}{3.6\times 10^{5}}\times N_{T}$$

where *N*_P_ and *N*_T_ represent the number of single‐target pairs needed and the number of targets assembled in each merged droplet. *t*_change_ can be expressed by

(5) $$t_{\mathrm{change}}=\frac{7}{60}\times \left(\mathrm{CEILING}.\mathrm{MATH}\left[\frac{N_{P}\times N_{T}}{3.6\times 10^{5}}\right]-1\right)+\frac{7}{60}\times \left(N_{T}-1\right)$$

where CEILING.MATH() is a function to round a number up to the nearest integer. Since the volume of large droplets is larger than that of the small droplets, the refill time for reinjecting large droplets is a bit more than that for reinjecting small droplets. To simplify, the reinjection time cost of small droplets was treated as equal to the time cost of large droplets, and *t*_refill_ can thus be calculated by

(6) $$t_{\mathrm{refill}}=\frac{4\pi \times {\left(\frac{D_{L}}{2}\right)}^{3}\times N_{P}\times N_{R}}{3\times 500\times 10^{9}}+\frac{10}{60}\times \left(N_{T}-1\right)$$

where *D*_L_ is the diameter of the large droplet, which equals 44.5 µm, and *N*_R_ represents the number of rounds of the reinjection process. For a two‐target assembly, *N*_R_ = 2. For a three‐target assembly, *N*_R_ = 2 + 2 + 1 = 5. Lastly, *t*_merge_ is given by

(7) $$t_{\mathrm{merge}}=\frac{N_{P}}{110\times 3600}\times \left(N_{T}-1\right)$$

For example, assuming that 5.4 × 10^5^ single‐target pairs were needed, for a two‐target assembly, the time cost (h) can be calculated as 15 / 60 + 5.4 × 10^5^ / (3.6 × 10^5^) × 2 + 7 × 3 / 60 + 5.4 × 10^5^ × 4π / 3 × (44.5 / 2)^3^ / (500 × 10^9^) × 2 + 10 / 60 + 5.4 × 10^5^ / (110 × 3600), which equals to 5.23 h. For a three‐target assembly, the time cost (h) can be calculated as 15 / 60 + 5.4 × 10^5^ / (3.6 × 10^5^) × 3 + 7 × 6 / 60 + 5.4 × 10^5^ × 4π / 3 × (44.5 / 2)^3^ / (500 × 10^9^) × 5 + 10 / 60 × 2 + 5.4 × 10^5^ / (110 × 3600) × 2, which equals to 8.76 h. The time it takes to generate other amounts of single‐target pairs can be deduced in this manner.

### Droplet‐Based Bead Immunoassay of IFN‐γ for Characterizing NK‐92MI and K562 Cells Interactions

Streptavidin‐coated fluorescent beads were conjugated with biotinylated anti‐IFN‐*γ* antibody (B133.5) by incubating 76.4 µL of beads with 2.31 µL of antibody in 25 µL of PBS at room temperature (R.T.) for 2 h. Anti‐IFN‐*γ* antibodies (2G1) were labeled with blue fluorescent Alexa Fluor 350 according to the manuals provided by the manufacturer. The capture antibody‐conjugated beads were mixed with 0.4 µL of Alexa Fluor 350 labeled detection antibodies in 200 µL of culture medium, and the mixture was used for the generation of single bead‐encapsulating droplets. NK‐92MI cells (ATCC, CRL‐2408) and K562 cells (ATCC, CCL‐243) were first stained with green and red fluorescence and then sorted. Subsequently, large droplets encapsulating single K562 cells were merged with droplets containing single beads and detection antibodies. The merged droplets were then combined with droplets encapsulating single NK‐92MI cells using another merging device. The collected droplets were placed in a 37 °C incubator for 12 h. The droplet‐based bead immunoassay was a homogeneous assay where the integrated fluorescence intensity in a droplet remained constant, but the peak fluorescence intensity increased in the presence of IFN‐*γ*. After co‐incubation, the collected droplets were subject to fluorescence‐based screening and sorted. The flow rates of the gapping oil and reinjected droplets were 1000 and 50 µL h^−1^, respectively, corresponding to a frequency of ≈150 Hz. Subsequently, the cells from both negative and positive droplets were extracted for RNA sequencing.

### Statistical Analysis

Statistical information, including sample size (n), the specific statistical test, data presentation, and the meaning of the significance symbol, was all included in relevant figure legends.

## Conflict of Interest

H.C. Shum is a scientific advisor of EN Technology Limited, MicroDiagnostics Limited, Upgrade Biopolymers Limited, and PharmaEase Tech Limited, in which he owns some equity; he is also a managing director of the research centre, Advanced Biomedical Instrumentation Centre Limited. The works in the paper are, however, not directly related to the works of these three entities, as far as we know.

## Supporting information



Supporting Information

Supplemental Movie 1

Supplemental Movie 2

Supplemental Movie 3

Supplemental Movie 4

Supplemental Movie 5

Supplemental Movie 6

Supplemental Movie 7

Supplemental Movie 8

Supplemental Movie 9

Supplemental Movie 10

## Data Availability

The data that support the findings of this study are available in the article and the supplementary materials of this article.
