# Supplementary material for: Single‐Target Pairing System (StarPair) for Large‐Scale Interrogation of Cell–Cell Interactions
Source: Adv Sci (Weinh). 2025 Dec 12;13(11):e13951. doi: 10.1002/advs.202513951 (PMC12931200; doi:10.1002/advs.202513951)
Supplement: Supplementary file 1 — Supporting Information [file ADVS-13-e13951-s008.pdf]

Supporting Information

Single-Target Pairing System (StarPair) for Large-Scale Interrogation of Cell-Cell Interactions

*Tianjiao Mao[1], Lang Nan[2], Miao Xu[1], Kehao Zeng[1], Yuchao Wang[1], Ziyu Han[3] and Ho Cheung Shum[1,3,4] **

[1] Department of Mechanical Engineering, The University of Hong Kong, Pokfulam Road, Hong Kong SAR, 000000, China.

[2] School of Instrument Science and Technology, Xi'an Jiaotong University, Xianning West Road, Xi'an, Shaanxi Province, 710049, China.

[3] Advanced Biomedical Instrumentation Centre, Hong Kong Science Park, Shatin, New Territories, Hong Kong SAR, 000000, China.

[4] Department of Chemistry and Department of Biomedical Engineering, City University of Hong Kong, Tat Chee Avenue, Kowloon, Hong Kong SAR, 000000, China.

[*] Correspondence should be addressed to Ho Cheung Shum. E-mail: ashum@cityu.edu.hk

This PDF file includes:

Supplementary text

Figures S1 to S18

Tables S1 to S3

Legends for Movies S1 to S10

Supplementary references

Supplementary Note 1. Culture and Sorting of Bacteria Cells

Lactiplantibacillus plantarum (CGMCC No.1258) were cultivated in MRS broth (69966, Millipore) to logarithmic growth phase (OD600 = 0.5). The bacterial cells were centrifuged and fluorescently labeled with BacLight Green (B-35000, Thermo Fisher Scientific) and BacLight Red (B-35001, Thermo Fisher Scientific) bacterial stains using a dye concentration of 5 μM in PBS for 30 min (RT). The bacterial cells were then resuspended with LB broth (L3522, Sigma-Aldrich) supplemented with 18 vt% opti-prep for fluorescence-based sorting.

Supplementary Note 2. Characterization of Secretion Level of IFN-*γ* Protein upon Co-Incubation of NK-92MI Cells and K562 Cells Using Bulk Sandwich Enzyme-Linked Immunosorbent Assay (ELISA)

Capture antibodies were prepared and immobilized on the fluorescent purple bead the same way as in bead-based immunoassay in droplets. Detection antibodies (M700A, Thermo Fisher Scientific) were conjugated with horseradish peroxidase (HRP) by HRP conjugation kit (ab102890, Abcam), according to manuals provided by the vendor. First, biotin-conjugated capture antibodies at 1 μg mL^{-1} were added to a streptavidin-coated plate (15125, Thermo Fisher, USA) and incubated for 2 h (R.T.). Excess antibodies were removed by washing each well three times with wash buffer (N503, Thermo Fisher Scientific). To prepare the standard solutions, the IFN-*γ* protein (300-02-20UG, Thermo Fisher Scientific) was solved with 20 μL of phosphate-buffered saline (PBS) and then diluted to different concentrations using a mixture of complete RPMI 1640 medium (61870036, Thermo Fisher Scientific) and NK-92MI specialized medium (CM-0533, Procell) at a volume ratio of 1:1. To acquire the IFN-*γ* proteins secreted by cells, 1 $\times 10^5$ NK-92MI cells were co-incubated with 1×10^5 K562 cells in 200 μL of culture medium in triplicate for 12 h; the cells were then centrifuged, and the supernatant was extracted. For the control group, a mixture of culture medium at equivalent volume was used. The above solutions were added to the plate and incubated for 2 h. After washing for 3 times, HRP-conjugated detection antibodies at 0.25 μg mL^{-1} were added and incubated for 1 h (R.T.). Washing was conducted carefully with wash buffer for 4 rounds and PBS for another round to remove all the unused detection antibodies. Next, HRP substrate (15159, Thermo Fisher Scientific) was added and incubated for 15 min, after which the stop solution was added to stop the reaction. Lastly,

OD values were measured at 570 nm using a microplate reader (SpectraMax iD3, Molecular Devices).

Supplementary Note 3. Validation of Droplet-Based Bead Immunoassay of Standard IFN-γ Proteins and Fluorescence-Based Screening System

For the validation of droplet-based bead immunoassay of standard IFN-γ proteins, droplet sizes were adjusted to the sizes of merged droplets combining three targets. In the IFN-γ (+) group, IFN-γ proteins at a concentration of 330 ng mL^{-1} (equal to the average IFN-γ level for NK-92MI and K562 cell co-incubation in bulk) were mixed with the Alexa Fluor 350 conjugated detection antibodies and capture antibody-conjugated beads for droplet generation. In the IFN-γ (-) group, Alexa Fluor 350 conjugated detection antibodies were mixed with capture antibody-conjugated beads but no IFN-γ proteins were added for droplet generation. After incubation at 37 °C for 2 h, droplets were reinjected into the sorting device for fluorescence screening. The flow rates of the gapping oil and reinjected droplets were 1000 and 50 μL h^{-1}, respectively. After determining the appropriate sorting threshold based on the fluorescence intensity profile of droplets in the IFN-γ (-) group, droplet sorting was conducted for both groups to validate the ability of the fluorescence-based screening system to enrich droplets concomitantly containing IFN-γ proteins, Alexa Fluor 350 conjugated detection antibodies, and capture antibody-conjugated beads.

Supplementary Note 4. Total RNA Purification and RNA-Sequencing

After co-incubation of NK-92MI cells and K562 cells in droplets for 12 h, IFN-γ positive and negative droplets were collected into two microtubes, respectively. 40 μL of PBS was first added, and 20 μL of 1*H*,1*H*,2*H*,2*H*-Perfluoro-1-octanol was then added into droplets to break the emulsion. The oil in the bottom was aspirated. The microtubes were centrifuged at 300 g for 5 min, and the supernatant was thoroughly removed. RNA purification was then performed using miRNeasy Tissue/Cells Advanced Kits (217684, Qiagen) according to the manufacturer's protocol with modified elution duration from 1 min to 3 min to obtain higher elution efficiency. For the two control groups, 10^6 NK-92MI cells and 10^6 K562 cells co-cultured and 10^6 NK-92MI cells cultured for 12 h were regarded as positive and negative controls, respectively. The

total RNA of these cells was then purified. RNA concentration and purity (A260/A280 ratio) were measured using NanoDrop™ 2000 Spectrophotometer (ND-2000, Thermo Fisher Scientific). Total RNA sequencing was performed by Novogene. The sequencing data were analyzed using NovoMagic.

Supplementary Note 5. qPCR Assays

Quantitative real-time RT-PCR of RNA extracted from positive and negative droplets after interactions between NK-92MI and K562 cells was performed using QuantiNova SYBR Green RT-PCR Kit (208154, Qiagen). Reaction mix was prepared according to the supplier's instructions. The specific primer sequences are: (1) IFNG: GAGTGTGGAGACCATCAAGGAAG (forward), TGCTTTGCGTTGGACATTCAAGTC (reverse); (2) TNF: CTCTTCTGCCTGCTGCACTTTG (forward), ATGGGCTACAGGCTTGTCACTC (reverse); (3) TNFRSF9: TCTTCCTCACGCTCCGTTTCTC (forward), TGGAAATCGGCAGCTACAGCCA (reverse); (4) BIRC3: GCTTTTGCTGTGATGGTGGACTC (forward), CTTGACGGATGAACTCCTGTCC (reverse); (5) CCL4: GCTTCCTCGCAACTTTGTGGTAG (forward), GGTCATACACGTACTCCTGGAC (reverse) (6) GAPDH: GTCTCCTCTGACTTCAACAGCG (forward), ACCACCCTGTTGCTGTAGCCAA (reverse). The RT-PCR thermal cycles were set as: 50 °C for 10 min; 95 °C for 2 min; 39 cycles of 95 °C for 5 s and 60 °C for 20 s.

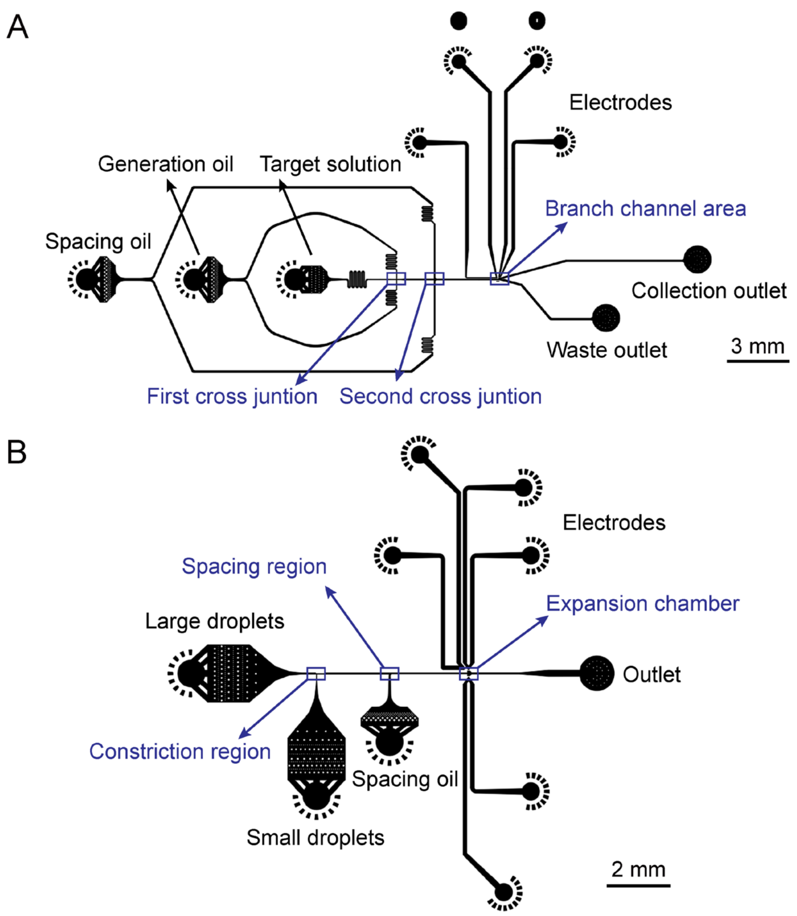

Figure S1. Schematic design of microfluidic devices. (A) Combinative droplet generation and sorting device. The height of the device is 43 µm. **(B)** Self-synchronization-based droplet pairing and merging platform. The height of the platform is 40 µm.

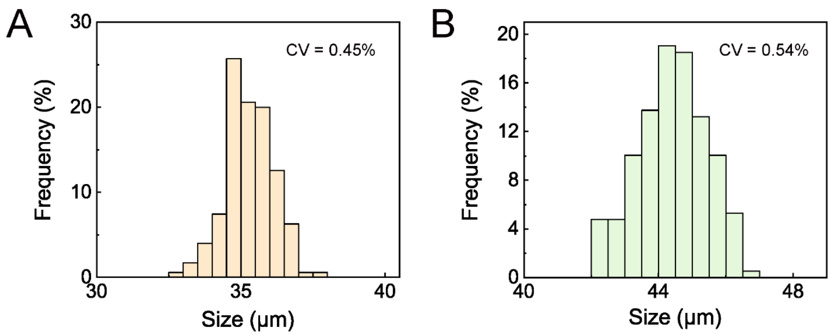

Figure S2. Size distribution of droplets. **(A)** Size distribution of small droplets when the flow rates of the dispersed phase, the first oil phase, and the second oil phase are set at 200, 1300, and 2200 μL h^{-1}, respectively. **(B)** Size distribution of large droplets when the flow rates of the dispersed phase, the first oil phase, and the second oil phase are set at 200, 500, and 2200 μL h^{-1}, respectively.

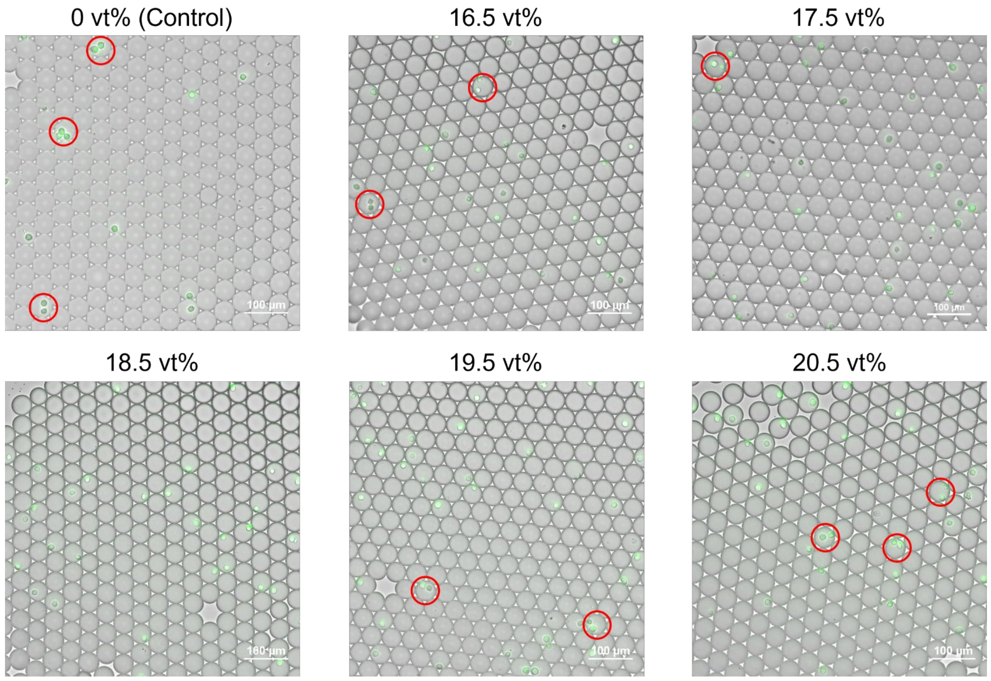

Figure S3. Microscope images of cell encapsulation in droplets at different OptiPrep concentrations. Droplets encapsulating more than a single cell are highlighted with red circles.

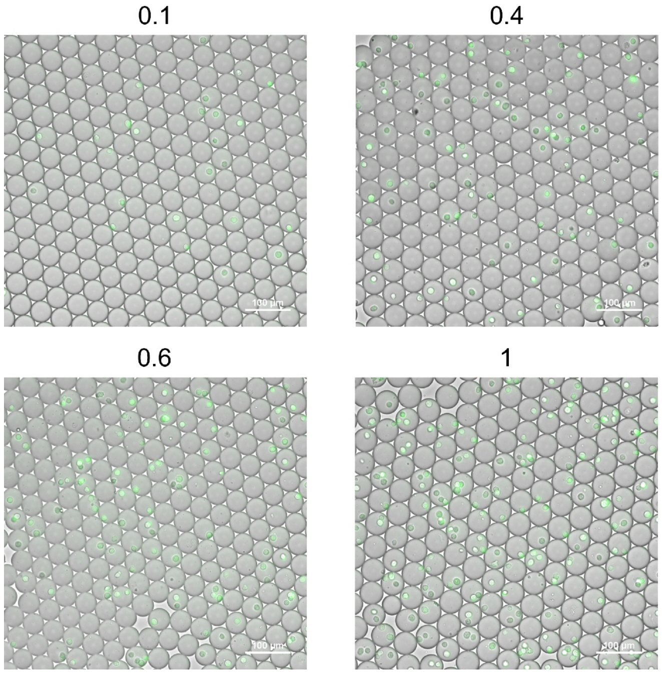

Figure S4. Microscope images of cell encapsulation in droplets at different cell concentrations (*λ*).

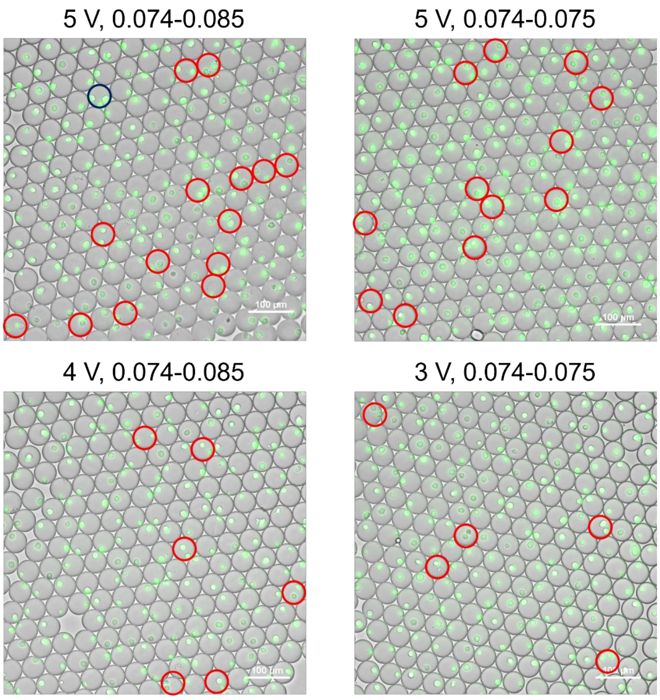

Figure S5. Microscope images of sorted droplets at inappropriate OptiPrep concentration (16.5 vt%) using different sorting parameters. Droplets encapsulating double cells and multiple cells are highlighted with red and dark blue circles, respectively.

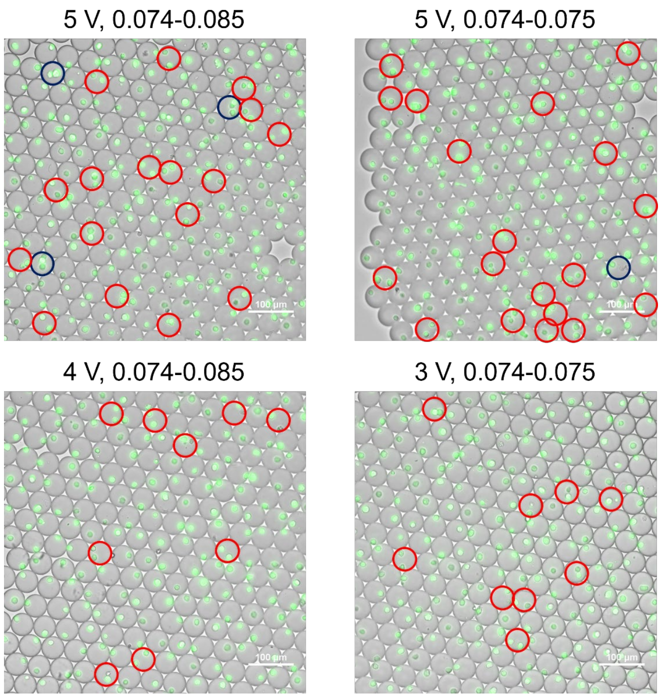

Figure S6. Microscope images of sorted droplets at inappropriate cell concentration (λ = 0.25) using different sorting parameters. Droplets encapsulate double cells and multiple cells are highlighted with red and dark blue circles, respectively.

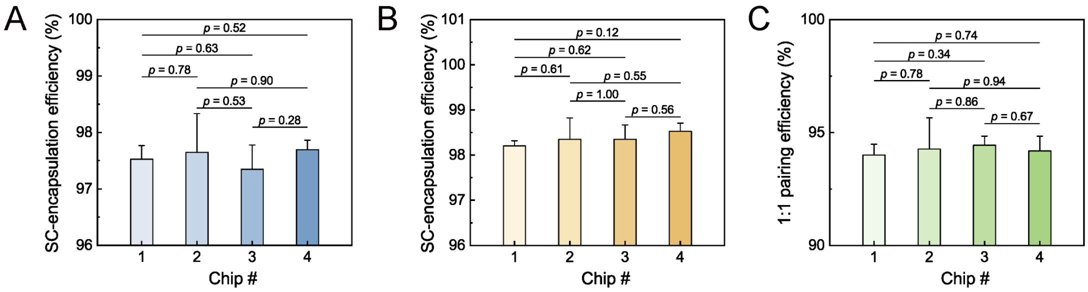

Figure S7. Chip-to-chip reproducibility evaluation. Single-cell (SC)-encapsulation efficiency of **(A)** small droplets and **(B)** large droplets after droplet generation and sorting using different chips. **(C)** One-to-one pairing ratio after droplet merging using different chips. Data are presented as mean ± SD. All the statistical analyses were done using Student's *t*-test.

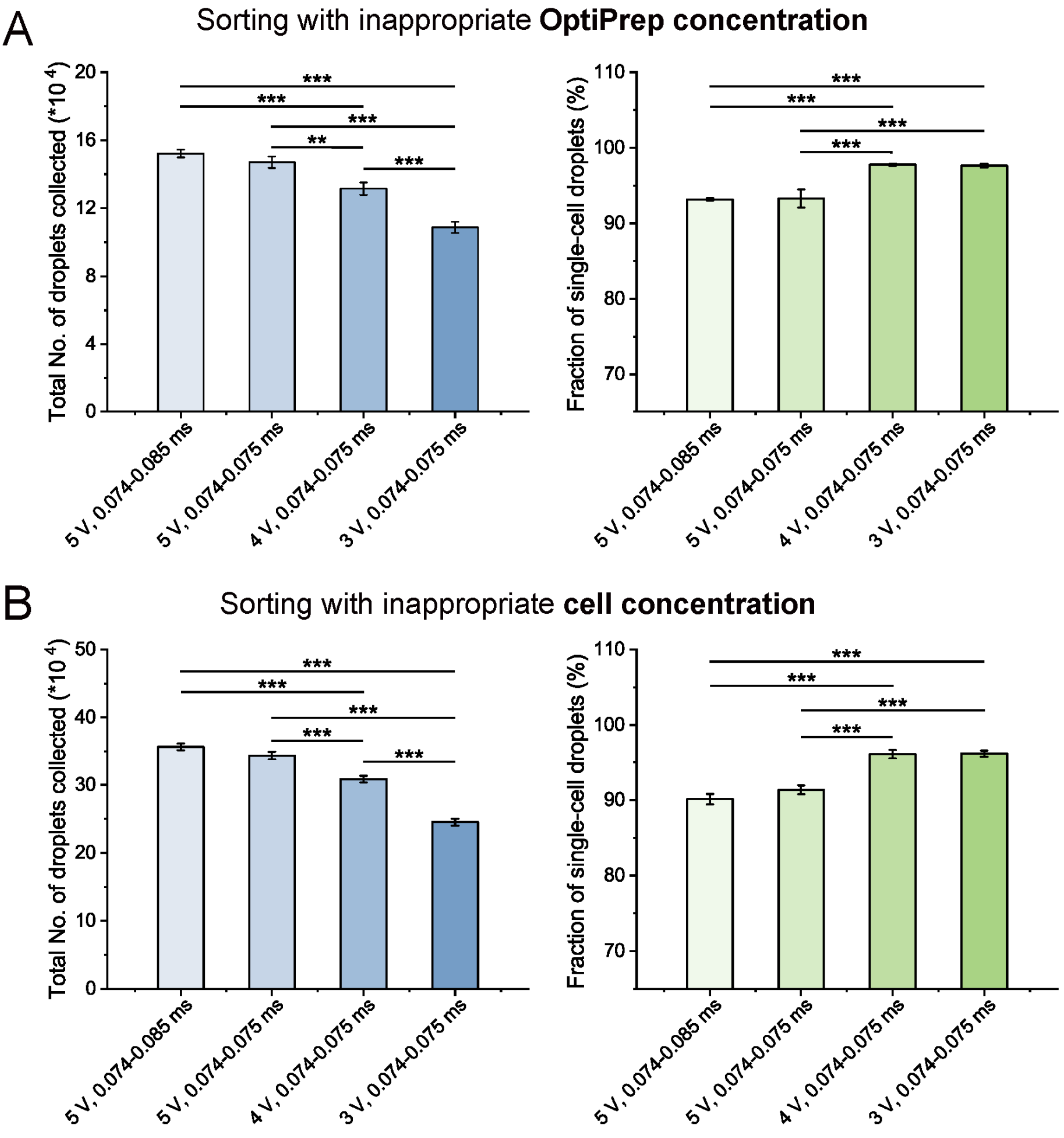

Figure S8. Impact of sorting parameters on sorting yield and purity. Total amount of droplets collected and fraction of single-cell droplets obtained when using different sorting parameter with **(A)** appropriate cell concentration (λ = 0.1) but inappropriate OptiPrep concentration ($C_{OptiPrep}$ = 16.5 vt%) where negative buoyancy occurs and **(B)** appropriate OptiPrep concentration ($C_{OptiPrep}$ = 18.5 vt%) but inappropriate cell concentration (λ = 0.25) where multiple-target droplets are generated. Data are presented as mean ± SD. Statistical comparisons were done using One-way ANOVA. "*", "**", and "***" represent that $p < 0.05$, $p < 0.01$, and $p < 0.001$, respectively. No significant difference is indicated if not annotated.

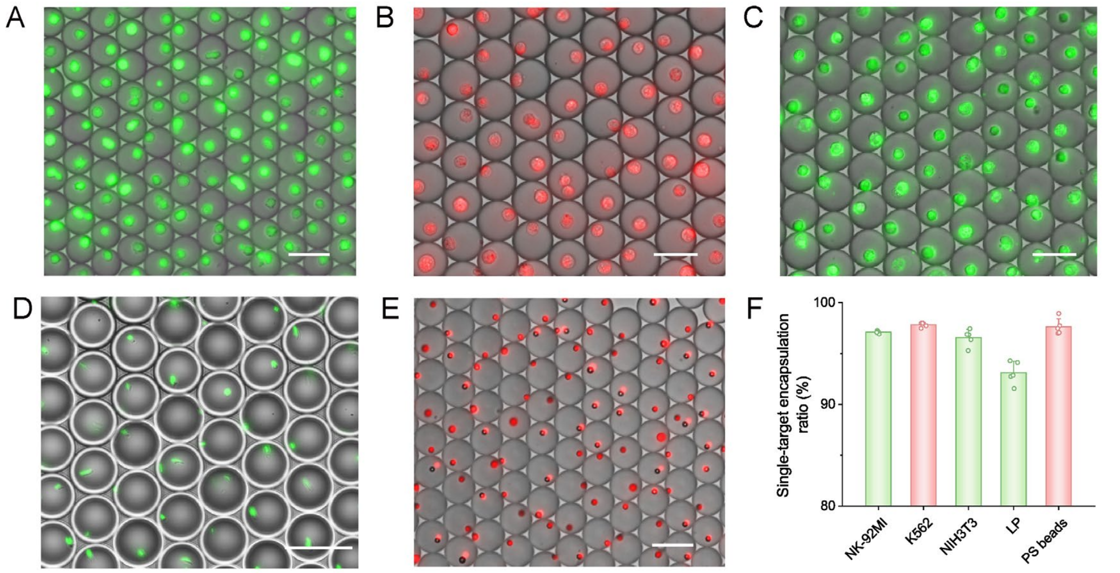

Figure S9. Precise single-target enrichment in droplets of diverse biological targets using StarPair. Photographs of droplets encapsulating single **(A)** NK-92MI cells, **(B)** K562 cells, **(C)** NIH3T3 cells, **(D)** *Lactiplantibacillus plantarum*, and **(E)** polystyrene beads. **(F)** Single-target encapsulation ratio for different targets. Scale bars: 50 μm.

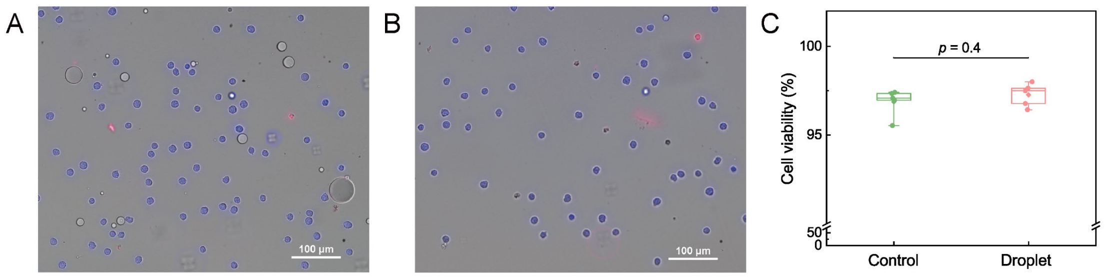

Figure S10. Cell viability evaluation. Microscope images of **(A)** cells subject to microfluidic manipulations and **(B)** cells cultured in flasks after live/dead staining. **(C)** Viability of cells in the control (cultured in flasks) and droplet (subject to droplet sorting and merging) groups. For the droplet group, droplets are demulsified 1 day post merging and the released cells are stained. Student's *t*-test was used to determine the difference in cell viability.

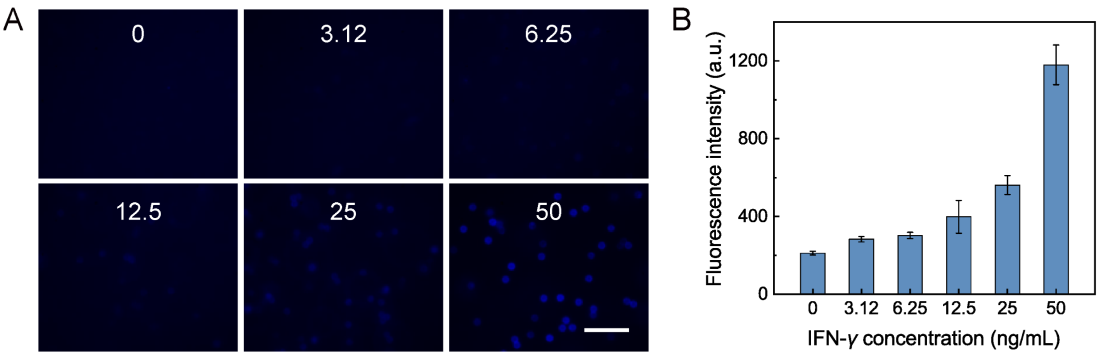

Figure S11. Validation of bead immunoassay of standard IFN-*γ* proteins in bulk. (A) Microscope images of beads at different concentrations of IFN-*γ* proteins (0, 3.12, 6.25, 12.5, 25, and 50 ng mL^{-1}). **(B)** Fluorescence intensity of beads at increasing concentrations of IFN-*γ* proteins. The bulk bead immunoassay is a heterogeneous assay where centrifugation and washing are involved after binding of IFN-*γ* proteins and binding of Alexa Fluor 350 labeled detection antibodies to remove background signals. Data are presented as mean ± SD. The limit of detection of the assay, determined as mean + 3SD at 0 ng mL^{-1}, is 1.28 ng mL^{-1}. At least 50 beads from three replicates are analyzed. Scale bar: 50 μm.

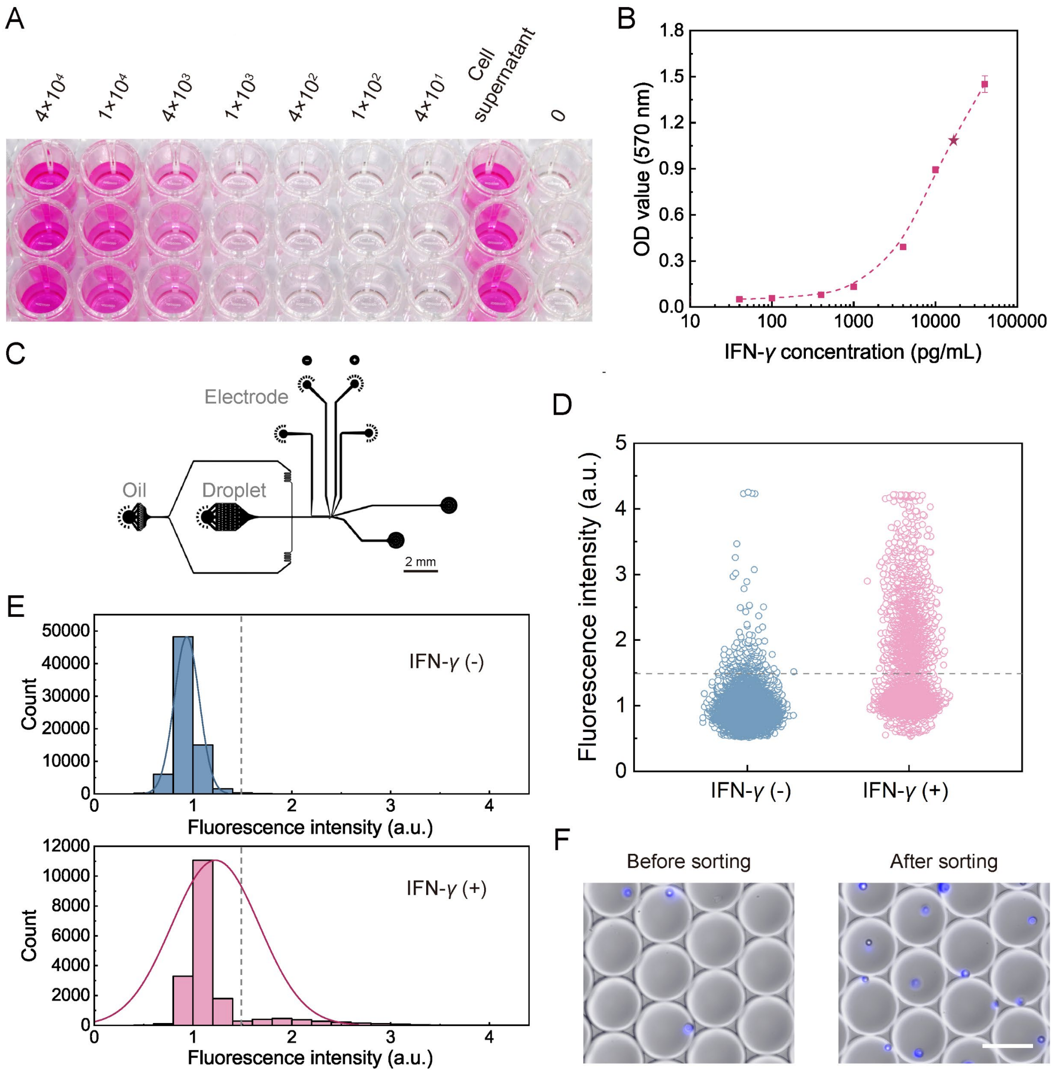

Figure S12. Validation of droplet-based bead immunoassay of standard IFN-γ proteins and fluorescence-based screening system. **(A)** Photograph of enzyme-linked immunosorbent assay (ELISA) wells tested with different concentrations of standard IFN-γ protein (40, 100, 400, 1000, 4000, 10000, and 40000 pg mL^{-1}) and cell supernatant from co-culture of 10^5 NK-92MI cells and 10^5 K562 cells. NK-92MI cells and K562 cells were co-cultured in 200 µL of cell culture medium in triplicate wells for 12 h before ELISA. 100 µL of supernatant was used in each ELISA well. "0" represents a negative control group without IFN-γ added. **(B)** Optical density (OD) value at different IFN-γ concentrations. OD values of the standard IFN-γ protein groups are shown as "■" and that of the cell supernatant group is shown as "★". **(C)** Schematic of the fluorescence-based screening system for detecting fluorescence signals of droplets and sorting desired droplets. The height of the device is 45 µm. **(D)** Fluorescence intensity of droplets. In the IFN-γ (+) group, droplets were generated by a mixture of IFN-γ proteins, Alexa Fluor 350 conjugated detection antibodies, and capture antibody-conjugated beads. In the IFN-γ (-) group , droplets were generated by a mixture of Alexa Fluor 350 conjugated detection antibodies and capture antibody-conjugated beads without adding IFN-γ proteins. **(E)** Histogram showing the distribution of fluorescence intensity of droplets in the IFN-γ (-) and the IFN-γ (+) groups. **(F)** Microscope images of droplets in the IFN-γ (+) group before and after

sorting. Only droplets concomitantly containing IFN-γ proteins, Alexa Fluor 350 conjugated detection antibodies, and capture antibody-conjugated beads could be sorted. The minimum sorting threshold indicated by the grey dashed line is determined as mean + 3SD of PMT voltage of droplets in the IFN-γ (-) group. Scale bar: 50 μm.

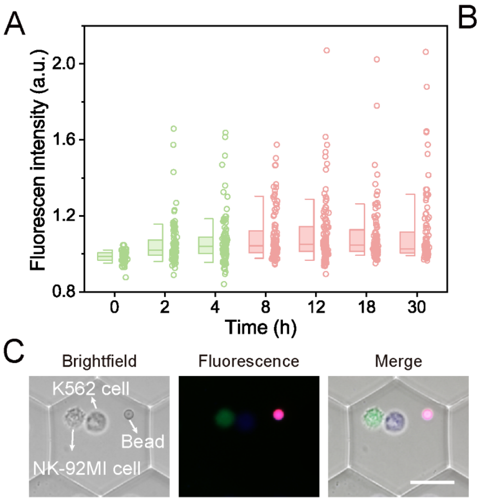

B

Group	*p* value
0 h, 2 h	7.24×10^{-7}
0 h, 4 h	1.47×10^{-7}
0 h, 8 h	9.29×10^{-11}
0 h, 12 h	4.09×10^{-9}
0h, 18 h	3.84×10^{-9}
0 h, 30 h	1.62×10^{-7}
2 h, 8 h	0.038
2 h, 12 h	0.012
2 h, 18 h	0.027
2 h, 30 h	0.029

Figure S13. Dynamics of interactions between NK-92MI and K562 cells. (A) Time evolution of the fluorescence intensity of beads after the assembly of cells and beads into droplets. N > 80 for each time point. The color shifting from green to red represents the period with increasing number of fluorescent beads changing to the period with stable number of fluorescent beads. **(B)** Calculated p values for groups of fluorescence intensities at different time points using Student's t-test. Groups not listed in the figure show no significant differences. **(C)** Photographs of an evaporated droplet with a bead showing blue fluorescence after co-incubation of cells for 12 h. The red fluorescence is emitted by the bead itself. Scale bar: 25 μm.

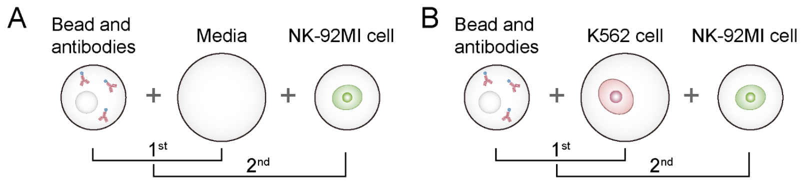

Figure S14. Schematic of assembling different targets to characterize interactions between NK-92MI and K562 cells. Droplets containing the bead and antibodies are first merged with **(A)** droplets containing pure media (NK-92MI group) or **(B)** droplets encapsulating single K562 cells (NK-92MI+K562 group), and then merged with droplets encapsulating single NK-92MI cells.

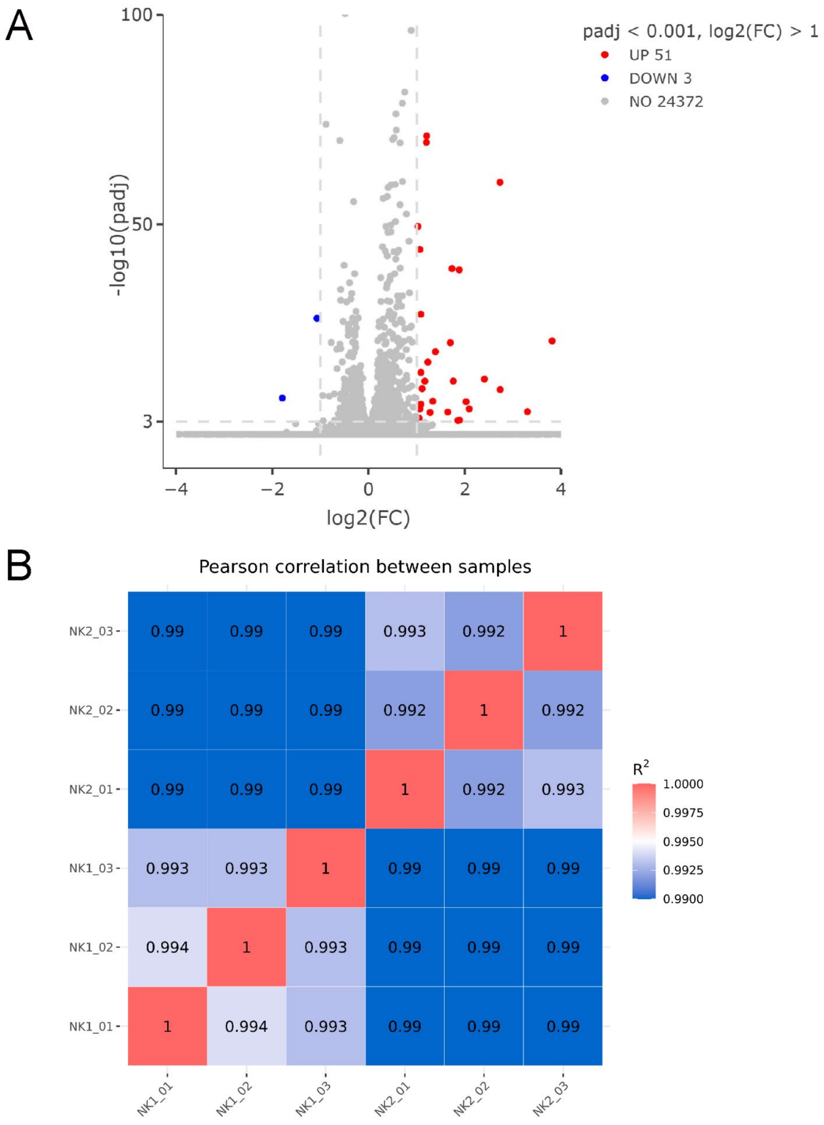

Figure S15. Comparison of the transcriptional profiles between the initially and eventually enriched NK-92MI cells in droplets. (A) Volcano plot for differential expression gene (DEG) analysis. Few DEGs are detected, indicating the overall transcriptional profile is not affected by droplet microfluidic manipulations. **(B)** Pearson correlation between two cell groups. “NK1_01”, “NK1_02”, and “NK1_03” stand for three replicates of initially enriched NK-92MI cells. “NK2_01”, “NK2_02”, and “NK2_03” stand for three replicates of eventually enriched NK-92MI cells. Great reproducibility is shown by $R^2 > 0.99$.

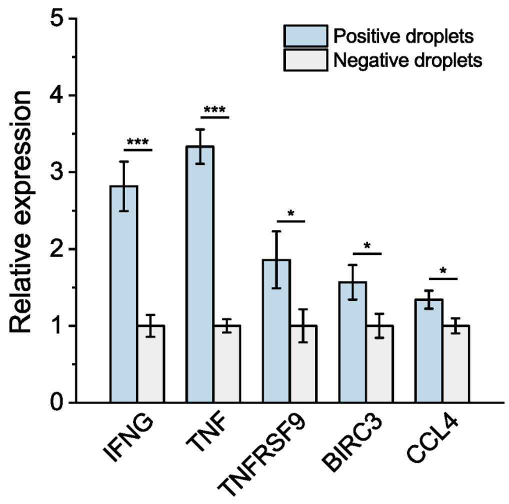

Figure S16. Relative expression of genes in positive and negative droplets after NK-92MI cell-K562 cell interactions. Data are presented as mean ± SD (n = 3). All the statistical analyses were done using Student's *t*-test. $*p < 0.05$ and $***p < 0.001$.

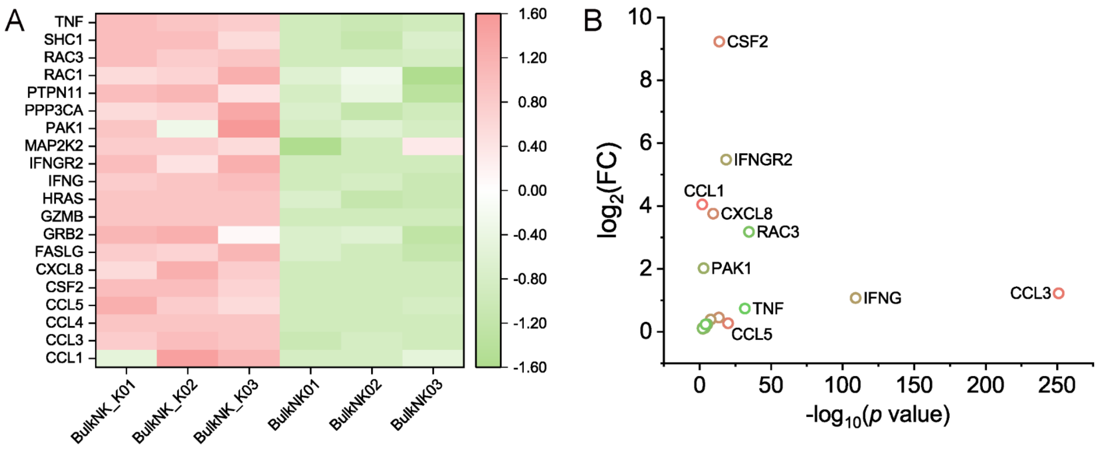

Figure S17. Transcriptomic profiles of NK-92MI cells upon bulk co-incubation with K562 cells. (A) Heatmap showing the gene expressions of natural killer activation-related genes obtained from RNA-sequencing. "Bulk NK_K" refers to the co-culture of NK-92MI and K562 cells, and "BulkNK" refers to pure NK-92MI cells. Three replicates are evaluated for each group. **(B)** Up-regulated gene expressions listed in Panel **(A)** comparing the "Bulk NK_K" group with the "BulkNK" group. For GZMB and CCL4, the *p* values approach 0, and $-\log_{10}(p$ value) is far off-axis. Their $\log_2(FC)$ equals to 1.46 and 1.61, respectively.

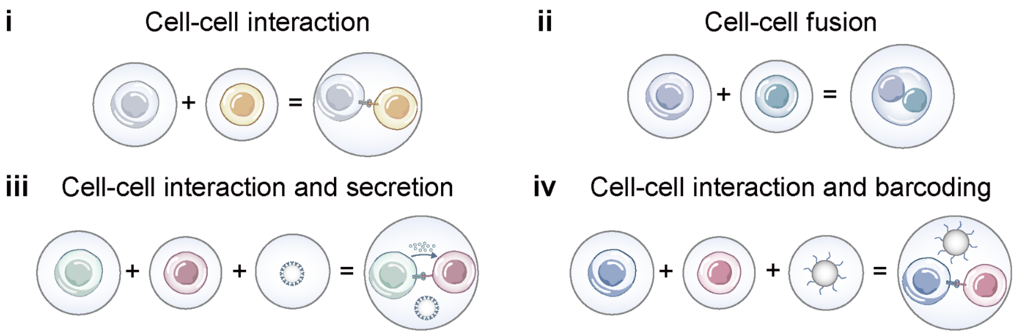

Figure S18. Schematic of various types of cell-cell interaction studies that can be enabled using StarPair.

Table S1. Summary of optimized operational parameters for single-target pairing using StarPair.

Set of steps	Type of operational parameters	Details
Droplet generation and sorting	Flow rates	(1) Small droplets: 200 : 1300 : 2200 μL h^{-1} (cell suspension : oil : spacing oil), corresponding droplet size: 35.7 μm;<br>(2) Large droplets: 200 : 500 : 2200 μL h^{-1} (cell suspension : oil : spacing oil), corresponding droplet size: 44.5 μm
	OptiPrep concentration	Depending on the target type: refer to Table S2
	Cell concentration	(1) Small droplets: $\lambda = 0.12$;<br>(2) Large droplets: $\lambda = 0.15$
	Sorting parameters	(1) Small droplets: 0.5-4.5 V, 0.074-0.075 ms for the last 10 μL of cells for each round of sorting, otherwise 0.5-5 V, 0.074-0.075 ms;<br>(2) Large droplets: 0.5-4 V, 0.074-0.075 ms for the last 10 μL of cells for each round of sorting, otherwise 0.5-5 V, 0.074-0.075 ms
Pairing and merging of pre-sorted droplets	Flow rates	(1) Two-target assembly: 20 : 35 : 200 μL h^{-1} (small droplets : large droplets : spacing oil);<br>(2) Three-target assembly: the first round: 20 : 35 : 200 μL h^{-1} (small droplets : large droplets : spacing oil); the second round: 20 : 55 : 300 μL h^{-1} (small droplets : large droplets : spacing oil)
	Electric voltages	(1) Two-target assembly: sine wave, 600 $V_{p\text{-}p}$, 30 kHz<br>(2) Three-target assembly: the first round: sine wave, 600 $V_{p\text{-}p}$, 30 kHz; the second round: sine wave, 650 $V_{p\text{-}p}$, 30 kHz

Note: The detailed values of sorting parameters here are applicable to SP2/0 cells. Because different types of cells exhibit different fluorescence intensity profiles even when using the same fluorescent dye for staining. These sorting parameters may need to be adjusted according to the fluorescence intensities of the input targets.

Table S2. Optimized OptiPrep concentrations for different biological targets.

Target type	OptiPrep concentration [vt%]
SP2/0 cells (ATCC, CRL-1581)	18.5
NK-92MI cells (ATCC, CRL-2408)	16.5
K562 cells (ATCC, CCL-243)	17
NIH3T3 cells (ATCC, CRL-1658)	18.5
293T cells (ATCC, CRL-3216)	17
A549 cells (ATCC, CCL-185)	18.5
Lactiplantibacillus plantarum (CGMCC No.1258)	18
Polystyrene beads	15

Note: For resuspension of cells and polystyrene beads, OptiPrep solution is mixed with cell culture medium supplemented with 10% fetal bovine serum (FBS) and 1% Penicillin-Streptomycin. For resuspension of bacteria, OptiPrep is mixed with MRS broth.

Table S3. Comparisons between StarPair and currently available systems for single-target pairing.

System	Pairing efficiency	Throughput [targets]	Multiplexing capability	Sample loss[b)]	System complexity
StarPair (our system)	Two-target: 95% Three-target: 92%	10^5-10^6	√	×	High
Microtrap-based systems[1-3]	Two-target: ~ 70%	10^2-10^3	×[a)]	√	Medium
Nanovial-based systems[4, 5]	Two-target: < 5%	10^5-10^7	×	×	Low
Droplet co-encapsulation systems[6, 7]	Two-target: < 5%	10^5-10^7	×	×	Low
Integrated droplet-based systems[8]	Two-target: 92% Three-target: 63%	10^3-10^5	√	√	High

[a)] Some microtrap-based systems are able to pair three targets but present low pairing efficiencies, and are therefore not included here.

[b)] Sample loss refers to the loss of input cells when excluding the pairing efficiency. In microtrap-based systems, the input target quantity is higher than the number of traps to increase the trapping efficiency. In integrated droplet-based systems, different batches of droplets are mixed in a pool and the target-encapsulating droplets are packed and merged in a fixed order, leading to the loss of other droplets containing targets.

Movie S1. Sorting of small droplets encapsulating single cells (SP2/0). The movie is recorded at 1000 fps and played at 5 fps.
Movie S2. Sorting of large droplets encapsulating single cells (SP2/0). The movie is recorded at 1000 fps and played at 5 fps.
Movie S3. Synchronization of two batches of reinjected single-cell encapsulating droplets in the constriction region. The movie is recorded at 1000 fps and played at 10 fps.
Movie S4. Separation of two batches of reinjected single-cell encapsulating droplets in the spacing region. The movie is recorded at 1000 fps and played at 10 fps.
Movie S5. Merging of two batches of reinjected single-cell encapsulating droplets in the expansion chamber. The movie is recorded at 1000 fps and played at 10 fps.
Movie S6. Merging of single K562 cell- and single bead-encapsulating droplets. The movie is recorded at 1000 fps and played at 10 fps.
Movie S7. Merging of droplets encapsulating single K562 cells and single beads and droplets containing single NK-92MI cells. The movie is recorded at 1000 fps and played at 10 fps.
Movie S8. Sorting of droplets encapsulating single beads with IFN-γ proteins but without cells (IFN-γ (+)). The movie is recorded at 1000 fps and played at 20 fps.
Movie S9. Sorting of droplets encapsulating single IFN-γ positive beads without IFN-γ proteins and cells (IFN-γ (-)). The movie is recorded at 1000 fps and played at 20 fps.
Movie S10. Sorting of droplet encapsulating single IFN-γ positive beads after co-incubation of NK-92MI and K562 cells. The movie is recorded at 1000 fps and played at 20 fps.

Supplementary References

[1] C. H. Wu, R. F. Chen, Y. Liu, Z. M. Yu, Y. W. Jiang, X. Cheng, "A Planar Dielectrophoresis-Based Chip for High-Throughput Cell Pairing", *Lab Chip* **2017**, *17* (23), 4008, https://doi.org/10.1039/c7lc01082f.

[2] B. Dura, S. K. Dougan, M. Barisa, M. M. Hoehl, C. T. Lo, H. L. Ploegh, J. Voldman, "Profiling Lymphocyte Interactions at the Single-Cell Level by Microfluidic Cell Pairing", *Nat. Commun.* **2015**, *6*, https://doi.org/10.1038/ncomms6940.

[3] A. M. Skelley, O. Kirak, H. Suh, R. Jaenisch, J. Voldman, "Microfluidic Control of Cell Pairing and Fusion", *Nat. Methods* **2009**, *6* (2), 147, https://doi.org/10.1038/nmeth.1290.

[4] D. Challa, J. de Rutte, C. Konu, S. Udani, J. Liang, P. j. Krohl, R. Rondon, K. Bondensgaard, D. Di Carlo, J. Watkins-Yoon, "Function-First Discovery of High Affinity Monoclonal Antibodies Using Nanovial-Based Plasma B cell Screening", *bioRxiv* **2024**, 2024.08.15.608174, https://doi.org/10.1101/2024.08.15.608174.

[5] M. Mellody, Y. Nakagawa, A. Arnheim, L. Shang, C. Soo, N. Tsubamoto, S. Taylor, S. Shastry, W. Luk, I. Morales, R. James, D. Di Carlo, "Sealable Capped Nanovials for High-Throughput Screening of Cell Growth and Function", *bioRxiv* **2025**, 2025.06.29.662236, https://doi.org/10.1101/2025.06.29.662236.

[6] S. Sarkar, P. Sabhachandani, D. Ravi, S. Potdar, S. Purvey, A. Beheshti, A. M. Evens, T. Konry, "Dynamic Analysis of Human Natural Killer Cell Response at Single-Cell Resolution in B-Cell Non-Hodgkin Lymphoma", *Front. Immunol.* **2017**, *8*, 1736, https://doi.org/10.3389/fimmu.2017.01736.

[7] M. A. Wheeler, I. C. Clark, H. G. Lee, Z. R. Li, M. Linnerbauer, J. M. Rone, M. Blain, C. F. Akl, G. Piester, F. Giovannoni, M. Charabati, J. H. Lee, Y. C. Kye, J. Choi, L. M. Sanmarco, L. Srun, E. N. Chung, L. E. Flausino, B. M. Andersen, V. Rothhammer, H. Yano, T. Illouz, S. E. J. Zandee, C. Daniel, D. Artis, M. Prinz, A. R. Abate, V. K. Kuchroo, J. P. Antel, A. Prat, F. J. Quintana, "Droplet-Based Forward Genetic Screening of Astrocyte-Microglia Cross-Talk", *Science* **2023**, *379* (6636), 1023, https://doi.org/10.1126/science.abq4822.

[8] J. L. Madrigal, N. G. Schoepp, L. F. Xu, C. S. Powell, C. L. Delley, C. A. Siltanen, J. Danao, M. Srinivasan, R. H. Cole, A. R. Abate, "Characterizing Cell Interactions at Scale with Made-to-Order Droplet Ensembles (MODEs)", *Proc. Natl. Acad. Sci. U. S. A.* **2022**, *119* (5), e2110867119, https://doi.org/10.1073/pnas.2110867119.
